# Modulation of the Electronic Properties of Co_3_O_4_ through Bi Octahedral Doping for Enhanced Activity in the Oxygen Evolution Reaction

**DOI:** 10.1021/acscatal.4c07911

**Published:** 2025-03-06

**Authors:** Damian Gorylewski, Filip Zasada, Grzegorz Słowik, Magdalena Lofek, Gabriela Grzybek, Katarzyna Tyszczuk-Rotko, Andrzej Kotarba, Paweł Stelmachowski

**Affiliations:** † Faculty of Chemistry, Institute of Chemical Sciences, Maria Curie-Sklodowska University, Maria Curie-Sklodowska Sq. 3, 20-031 Lublin, Poland; ‡ Faculty of Chemistry, Jagiellonian University, Gronostajowa 2, 30-387 Krakow, Poland

**Keywords:** electrocatalysis, water electrolysis, oxygen evolution reaction, cobalt spinel, bismuth doping, density functional theory

## Abstract

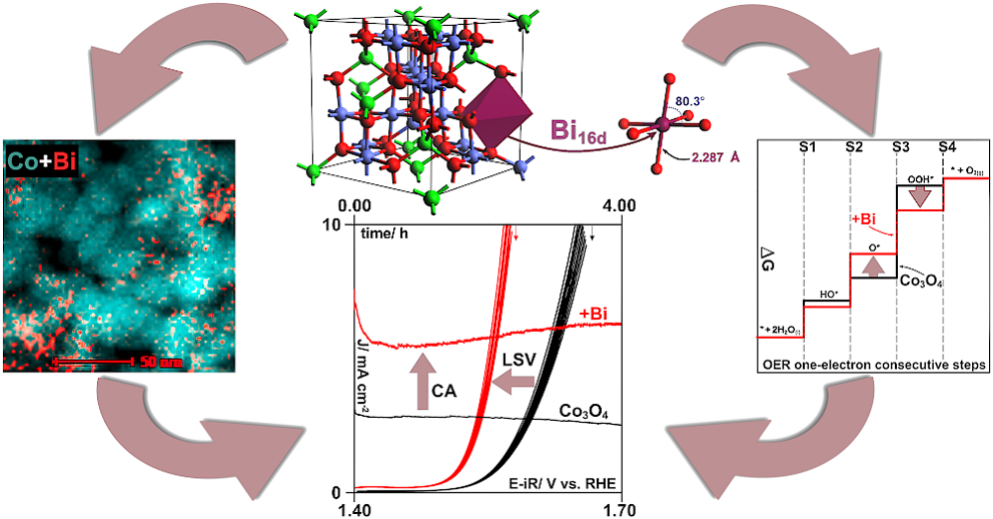

Developing a highly active and stable electrocatalyst for the oxygen evolution reaction (OER) is essential for efficient hydrogen production through anion exchange membrane water electrolysis powered by renewable electricity. Recently, there has been a renewed interest in designing electrocatalysts based on their work function optimization. The insights into the materials’ electronic properties gained from developing other heterogeneous catalysts, such as those used for N_2_O decomposition, can be thus leveraged to enhance the performance of the OER electrocatalysts. Knowing that Bi enhances the catalytic activity of Co_3_O_4_ in N_2_O decomposition, where the surface electronic properties play a crucial role, we hypothesized that it might also improve the electroactivity of the OER electroactivity. Therefore, we synthesized Bi-doped Co_3_O_4_ with different bismuth contents and studied the sample with a complementary set of physicochemical, electrochemical, and computational techniques. We found that promoting Co_3_O_4_ with atomically dispersed bismuth enhances its OER electrocatalytic properties by reducing the energy of the potential-determining step and improving electron charge transfer properties. Bismuth atoms enter octahedral sites in Co_3_O_4_, creating Bi active centers and enhancing the activity of vicinal Co sites in the OER. The Bi and modified Co centers are characterized by increased binding energy of the intermediate state of the metal–oxygen intermediate and increased density of states at the Fermi level. The former reduces the overpotential required for the OER, whereas the latter improves the reaction kinetics by decreasing the charge transfer resistance.

## 1 Introduction

The evolution of new technologies increases the electricity demand. Conventional power plants utilize fossil fuels and contribute to carbon dioxide emissions that are of severe public concern. Hydrogen is considered a game-changer in worldwide energy management and is starting to play a vital role in future mobile energy conversion devices.^1−3^ However, a kinetically limited anodic oxygen evolution reaction (OER) hinders water electrolysis for H_2_ production. The solution is to use an appropriate catalyst to reduce overpotential and provide high current density for the inherently slow OER. The effective catalysts should also be synthesized from abundant and cheap reagents.^1,2,4,5^

Among many materials, transition metals such as Fe, Co, and Ni are widely investigated as OER catalysts but suffer stability problems under acidic conditions. Co_3_O_4_-based catalysts are efficient, cost-effective, and easy to synthesize. They could potentially lower the entry threshold of renewable energy systems, making them more available, especially for large-scale applications. Importantly, they are stable in an alkaline environment and can be used in anion exchange membrane (AEM) electrolyzers.^1,6−9^

Ni-based (oxy)hydroxide NiO_*x*_H_*y*_ catalysts are gaining importance due to their low overpotential equal to ∼300 mV@10 mA cm^–2^ current density in an alkaline environment. Additionally, its adsorption energy satisfies the Sabatier principle. Moreover, the electrocatalytic activity of bare NiO_*x*_H_*y*_ can be enhanced due to doping with iron ions, which can replace the Ni atoms and decrease the OER overpotentials.^10−12^ However, it should be noted that a high level of iron content in the NiOOH leads to structural disorder and promotes the iron oxide hydroxide (FeOOH) segregation, drastically reducing OER activity.^10^ Therefore, it is essential to precisely dope this element, which can sometimes be a rather complicated and time-consuming task. According to the studies presented by Lee et al.,^12^ iron ions can be introduced into the γ-NiOOH structure by metal dissolution electrodeposition (MDE), which involves a dissolution of nickel substrate in the electrolyte containing Fe^3+^ ions and followed by the redeposition of dissolved species. The role of Fe^3+^ ions is to stabilize the Ni^2+^ and Fe^2+^ depositions, which are characterized by low reduction potentials. As a result, the catalyst adhesion with etched Ni substrate is enhanced. Moreover, MDE facilitates the formation of amorphous NiOOH phases, which can be crystallized during the Fe deintercalation–activation process, forming the NiOOH catalysts with integrated iron atoms. The fine-tuned Fe-γ-NiOOH catalyst is characterized by a low overpotential of 257 mV at a high current density of 100 mA cm^–2^, low Tafel slope of 49.74 mV dec^–1^, low charge transfer resistance of 551 mΩ, and high active surface area of 13.23 mF cm^–2^.^12^ Nevertheless, Fe- and Ni-based catalysts are prone to corrosion in alkaline solutions, which makes it unclear whether the catalytic surface components are the pristine catalysts or their transformation products. The corrosion also limits the potential catalyst service life.^13^

Cobalt oxides are more stable in alkaline conditions than Fe and Ni. Thus, they are often subjected to modifications with other metals to improve OER activity by modifying their electronic configuration and suitably adapting it for electrode reaction.^2,14−19^ Literature reports describe different ways to promote Co_3_O_4_ using noble metals, e.g., Ru, Rh, Pd, and Ag,^2,15,16,18,19^ sometimes with the use of other modifiers to enhance further the activity such as Ni foam and providing P-doping^15^ or utilizing carbon nanotubes, etc.^2^ Zhao et al.^16^ showed a new ruthenium-doped Co_3_O_4_ catalyst for the OER for which they achieved 118 mV decrease in the overpotential measured at 10 mA cm^–2^ (249 mV) compared to bare Co_3_O_4_ (367 mV). The authors argue that Rh single atoms play a crucial role in adjusting the electronic properties of surrounding Co atoms, altering bond energies between intermediates and active sites but that the catalytic function of individual ruthenium atoms was not crucial. Another example of an M-Co_3_O_4_ catalyst was presented by Zhang et al.,^2^ where Ag-doped Co_3_O_4_ deposited on carbon nanotubes exhibited 236 mV overpotential at 10 mA cm^–2^ current density (80 mV lower compared to the undoped Co_3_O_4_/CNT). They have shown that incorporating Ag atoms into the Co_3_O_4_ lattice facilitates the adsorption of *OH and *O onto the surrounding Co atoms. Additionally, Ag atoms participate in the *OOH adsorption process through the Ag–Co bridge. They concluded that as a result, the desorption of O_2_ is hindered and reaction barriers are reduced, which improves the kinetics of the oxygen evolution reaction.

Remarkable catalytic properties characterize the catalysts described above, but their synthesis involves expensive noble metals (Rh, Ag). An alternative concept of using base metals doped into the structure of Co_3_O_4_ to modify its electronic properties involves elements easily introduced into the cobalt spinel crystal lattice, such as Cr, Mn, Fe, Ni, Cu, or Zn.^14,20−26^ Zhang et al.^26^ incorporated Mn into the Co_3_O_4_ structure without manganese oxide segregation (Mn_0.8_Co_2.2_O_4_/NF), which shows 231 mV overpotential at the current density 10 mA cm^–2^, 41 mV lower than Co_3_O_4_/NF. Authors claimed that Mn-doping caused a specific disorder in the structure of Co_3_O_4_/NF, which was proportional to the Mn/Co ratio in the catalyst. Another example is reported by Behera et al.,^25^ where authors demonstrated 5% Cu–Co_3_O_4_ showing activity with 360 mV overpotential measured at the 10 mA cm^–2^ current density (ca. 110 mV lower vs bare Co_3_O_4_). However, for Cu > 5%, CuO segregation occurs on the catalyst surface. These studies revealed that introducing Cu into the Co_3_O_4_ structure tunes the electronic structure, improving, e.g., occupancy and, as a result, regulating the spin state presented at the octahedral site in the bulk-adjacent Co^3+^ ions. DFT studies confirmed these conclusions, indicating that it affects the binding strength of the oxygen intermediates, which are crucial in the rate-determining steps of the oxygen evolution and reduction reactions. Vazhayil et al.^21^ obtained hierarchically nanostructured Co_3_O_4_ doped with Ni, Mn, and Zn. Among the explored modifications, Ni–Co_3_O_4_ showed the highest OER activity of 10 mA cm^–2^ at an overpotential of 380 mV, outperforming bare Co_3_O_4_. Similarly, Zhang et al.^27^ introduced a fine-tuned Ni_0.1_Co_2.9_O_4_ catalyst, which showed the smallest overpotential of 349 mV compared with other Co_3_O_4_-based catalysts (more than 375 mV) and RuO_2_ (401 mV).

It was proposed already in the 80s that factors contributing to electrocatalysis could be divided into two main categories that simultaneously contribute to the catalytic mechanism of the OER: electron transfer and strength of the M-O bond. The work function is an important factor for electrocatalysis related to electron transfer.^28^ Recently, the design of electrocatalysts based on their electronic properties, including work function, has gained significant renewed attention. For example, it has been shown that in a series of Co–Mn oxides, the catalytic performance of the as-prepared samples is linearly dependent on the work function, indicating that the accelerated charge transfer process dramatically promotes OER activity.^29^ The latest advancements in work function-guided electrocatalyst design for diverse electrochemical energy applications have been comprehensively summarized in ref (30). Therefore, the knowledge gained in the work-function-based development of other heterogeneous catalysts can be leveraged to improve the OER electrocatalysts. One such reaction is the low-temperature N_2_O decomposition, in which the optimization of Co_3_O_4_-based catalysts is most effectively achieved using the work function parameter.^31^

In summary, many attempts have been made to dope cobalt spinel with non-noble metal ions, but most involved atoms with relatively low atomic numbers.^14,20−26^ On the other hand, promotion with heavy, noble metals yields the most active OER catalysts. The introduced heteroatoms may constitute a new active center (Cu^25^) or modify the adjacent cobalt ions (Rh^16^). Inspired by the excellent catalytic properties of atomically dispersed Bi-doped Co_3_O_4_ in the deN_2_O reaction,^32^ where it was shown to improve the oxygen recombination step crucial also in the case of the OER, it became the object of our research. For the first time in the reported literature, we aimed to utilize Bi-doped Co_3_O_4_ as a catalyst for the oxygen evolution reaction in an alkaline environment. Although the Bi position in the periodic table indicates that it has the strongest metallic properties in the group, it is not one of the most conductive metals, both electrically and thermally.^33^ Nevertheless, a significant advantage of Bi is its negligible toxicity. The study aimed to verify the hypothesis that the Bi doping of Co_3_O_4_ enhances its electrocatalytic activity in the oxygen evolution reaction through beneficial modifications of the electronic properties of cobalt spinel and to elucidate its specific role through a computational approach.

## 2 Materials and Methods

The research scheme is presented in the Supporting Information (Figure S1).

### 2.1 Synthesis of Bi_*x*_Co_3–_*_x_*O_4_ Catalytic Materials

Catalysts were synthesized by classical precipitation to obtain mixed oxides with the Bi_*x*_Co_3–*x*_O_4_ general formula, where *x* = 0, 0.02, 0.04, 0.07, 0.08, 0.11, and 0.19. The appropriate masses of Bi(NO_3_)_3_ × 5H_2_O (POCH, 99.99%) and Co(NO_3_)_2_ × 6H_2_O (Dor-chem, 99.99%), corresponding to the *x* value (see Table S1 and Section S1.1 in Supporting Information), were dissolved in a 2 mol L^–1^ HNO_3_ (POCH, PA) aqueous solution. This process was carried out to prepare a total volume of 15 mL of bismuth and cobalt solution, each with a cation concentration of 1 mol L^–1^. Then, the appropriate volume of 1 mol L^–1^ (NH_4_)_2_CO_3_ (Chempur, PA) was gradually added to the solutions until pH = 8.2 was reached to coprecipitate bismuth and cobalt hydroxides. The HLP 5 system deionized water was utilized in all experiments (Hydrolab, Poland). Acquired sediments were filtered on a Buchner funnel and washed with water to neutralize the pH. Next, obtained samples were dried within 12 h at 60 °C and then calcined on air for 4 h at 500 °C, with a temperature increase of 4 °C min^–1^.

### 2.2 Physicochemical Characterization

The samples were characterized in terms of their elemental composition (XRF), structure (XRD, Raman spectroscopy), and specific surface area (*S*_BET_). The microscopic imaging was performed using a transmission electron microscope equipped with a high-angle annular dark-field (HAADF) detector and an energy-dispersive X-ray spectrometer. The electronic properties of cobalt spinel catalysts doped with bismuth have been investigated by the determination of the work function (Φ) using the Kelvin method. Experimental details are described in the Supporting Information (SI), Section S1.2.

### 2.3 Electrochemical Measurements

OER activity and stability studies were performed using a 3-electrode system with a rotating disc glassy carbon electrode (RDGCE) as a working electrode, Pt wire as a counter electrode, and Hg/HgO as a reference electrode. The catalyst loading on the RDGCE was 200 μg cm^–2^. For the working electrode with a diameter of 3 mm and a geometric surface area of 0.07065 cm^2^, 0.014 mg of the catalyst was present on its surface. To determine the kinetic parameters of the oxygen evolution reaction, chronoamperometric measurements were performed in nine 15 min steps with potential changes from 1.43 to 1.83 V vs RHE with a 50 mV step. CV measurements in the potential range from 0 to 0.635 V vs Hg/HgO for ν = 5 mV s^–1^ were carried out for each sample before and after OER activity tests to examine the oxidation (A_1_ and A_2_) and corresponding reduction (C_1_ and C_2_) peaks originating from Co^2+^/Co^3+^ and Co^3+^/Co^4+^ redox transformations.^34^ Additional electrochemical characterization was performed using a K_3_[Fe(CN)]_6_/K_4_[Fe(CN)]_6_ redox system. Experimental details are described in the Supporting Information, Section S1.3.

### 2.4 Molecular Modeling and Construction of OER Free Energy Diagrams

Density functional theory with Hubbard-corrected functionals (DFT+*U*) was employed for all calculations using the Vienna Ab initio Simulation Package (VASP).^35^ The projector augmented wave (PAW) method was used to describe electron–ion interactions^36^ alongside the generalized gradient approximation with the PBE exchange functional.^37^ A standard Monkhorst–Pack grid was utilized,^38^ with a 5 × 5 × 5 sampling mesh for bulk and a 3 × 3 × 1 mesh for slab calculations, respectively. The cutoff energy was set to 450 eV, and a Methfessel–Paxton smearing^39^ with a σ parameter of 0.1 eV was applied. For solving the Kohn–Sham self-consistent field (SCF) equations, a convergence energy change of 10^–5^ eV between successive iterations was enforced.

For the construction of OER free energy diagrams, the methodology developed by Nørskov et al. was employed^40−42^ with the assumption that the overall process occurs in four single-electron steps, involving water splitting at the electrocatalyst active site, with the formation of an HO* intermediate (**step-1**), its subsequent oxidation to O* (**step-2**), followed by water dissociation atop O* (formation of OOH*, **step-3**), and the final step of the oxygen evolution (**step-4**). The electronic energies (Δ*E*^DFT^) of the above reaction steps were calculated by using the developed slab models exposing the (100) surface of cobalt spinel (Figure S12, SI). The free energies were calculated by employing entropic corrections computed using statistical thermodynamics, incorporating the approximations proposed by Man et al.^40^ with the computational hydrogen electrode used as a reference. Further calculation details are described in the Supporting Information, Section S5.

## 3 Results and Discussion

The materials prepared were investigated regarding their physicochemical properties as catalysts for the anodic oxygen evolution reaction. The results showing Bi content theoretically assumed in the synthesis protocol and instrumentally measured values, as well as the general formula used in this paper for the description of the samples, are presented in Table 1. All of the Bi-doped Co_3_O_4_ catalysts were tested in the N_2_O decomposition reaction to confirm the reactivity trend reported previously,^32^ and activity changed similarly to the reported values in dry and wet conditions, as shown in Figure S2. The deN_2_O reaction was used as a benchmark for changes in the electronic properties of the Bi–Co_3_O_4_ materials. Next, the samples were more thoroughly characterized and tested as electrocatalysts in the OER. The research scheme is presented in Figure S1.

**Table 1:** XRF Quantification Results of Bi-Doped Co_3_O_4_

theoretical Bi content (wt %)	real Bi content (wt %)	*x*	theoretical formula (Bi*_x_*Co_3–*x*_O_4_)
0	0	0	Co_3_O_4_
1.7	1.7	0.02	Bi_0.02_Co_2.98_O_4_
3.4	3.4	0.04	Bi_0.04_Co_2,96_O_4_
5.0	5.8	0.07	Bi_0.07_Co_2.93_O_4_
6.6	6.6	0.08	Bi_0.08_Co_2.92_O_4_
8.2	8.9	0.11	Bi_0.11_Co_2.89_O_4_
15.4	14.7	0.19	Bi_0.19_Co_2.81_O_4_

**Figure 1:**
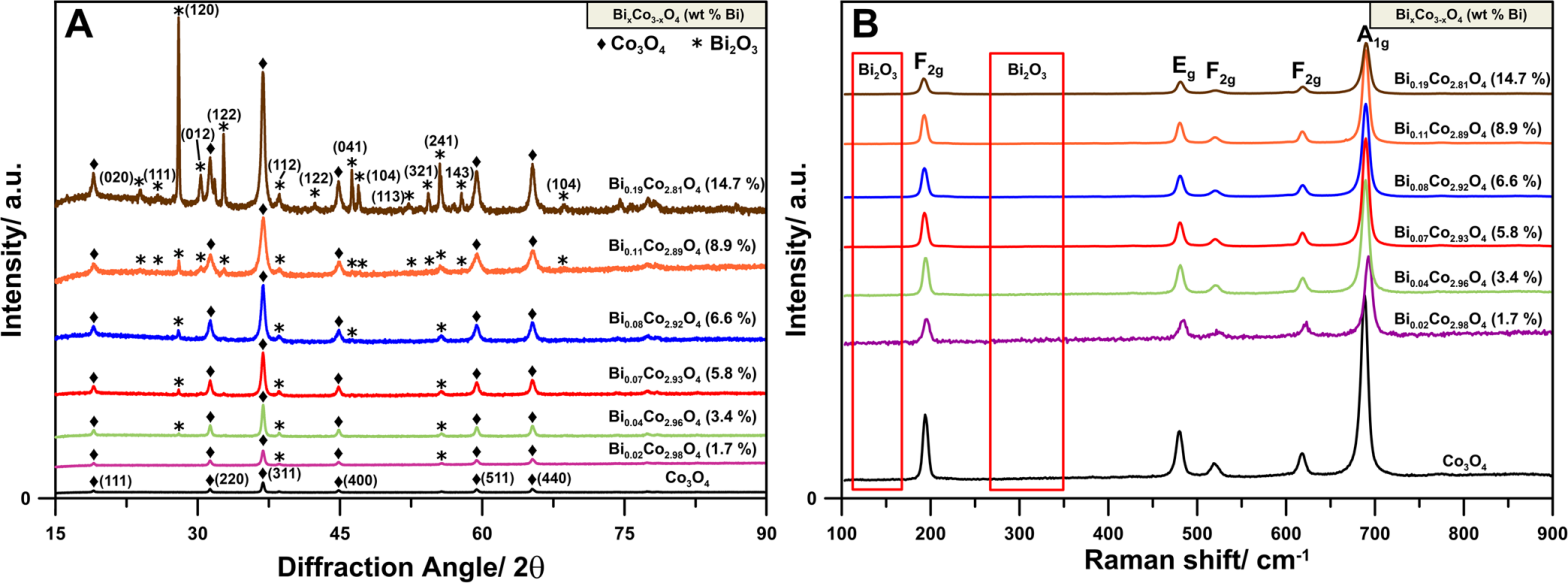
(A) X-ray diffraction patterns of Bi_*x*_Co_3–_*_x_*O_4_ samples. (B) Raman spectra of Bi_*x*_Co_3–_*_x_*O_4_ samples.

Although bismuth has only a slightly larger atomic radius than cobalt,^43,44^ it has a substantially higher ionic radius.^45^ Thus, its successful incorporation into the spinel matrix must be established. X-ray diffraction patterns reveal that even the smallest amount of Bi doping results in the segregation of the Bi_2_O_3_ phase (Figure 1A). However, Raman spectroscopy does not show the presence of Bi_2_O_3_ (Figure 1B).

**Figure 2:**
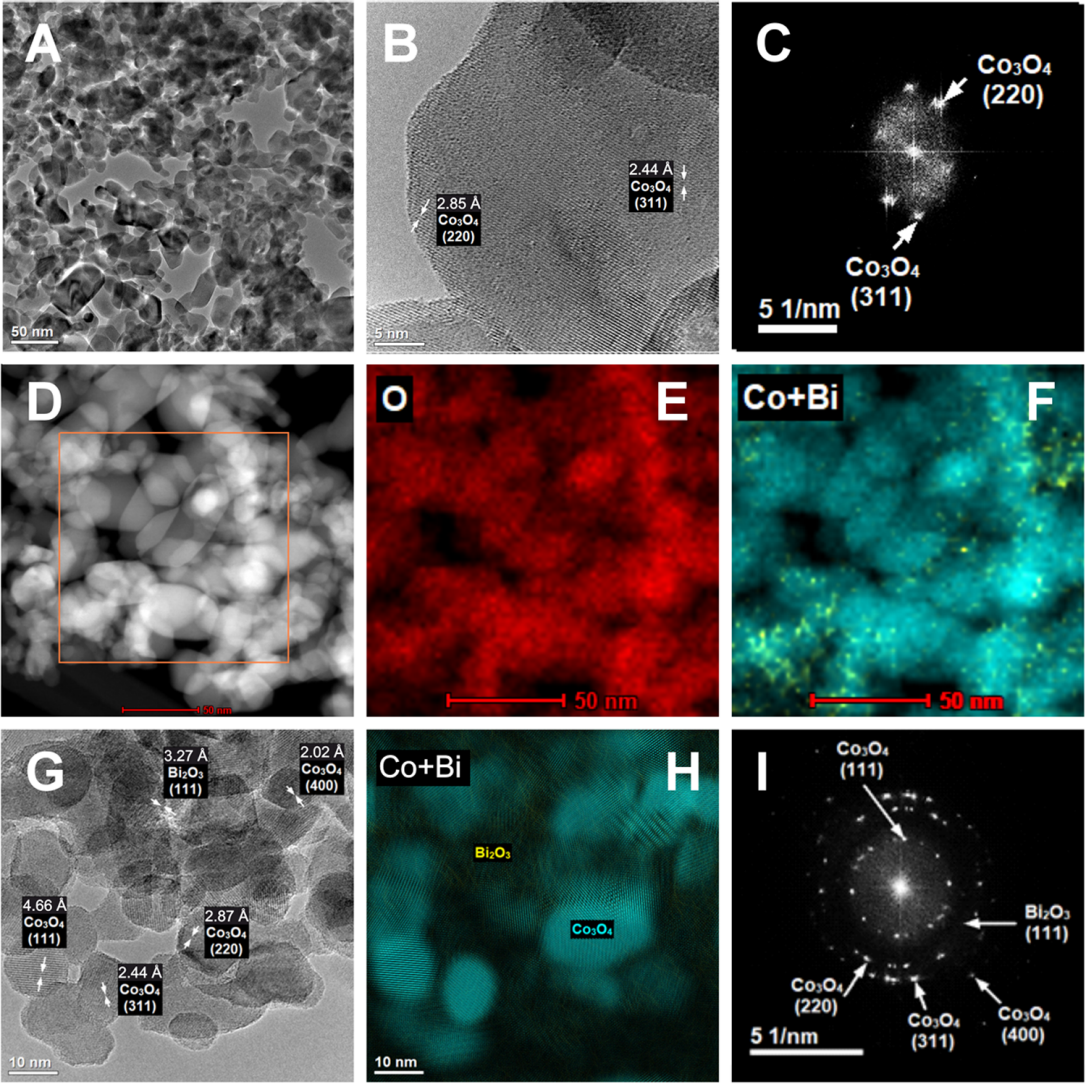
HR-TEM images and FFT patterns of the Bi_0.07_Co_2.93_O_4_ sample. (A) Low magnification overview, (B) high magnification view, and (C) associated FFT pattern. (D) A low magnification area was selected for elemental STEM-EDS analysis and associated (E) oxygen mapping and (F) cobalt and bismuth mapping. (G) High magnification area selected for elemental STEM-EDS analysis, associated (H) cobalt and bismuth mapping, and the (I) FFT pattern.

Analysis of all TEM images and STEM-EDS studies for the Bi_*x*_Co_3–*x*_O_4_ sample with *x* = 0.07 (Figures 2 and S3) shows very little bismuth. The HR-TEM images (Figure 2A,B,G) do not show isolated, well-defined crystallites of Bi_2_O_3_, only Co_3_O_4_ crystallites (Figure 2C,H). Bismuth in the sample is very well dispersed at the atomic or multiatomic level, as visualized in the EDS maps (Figure 2F,H). As shown in Figure 2I, no specks for Bi_2_O_3_ (111) are present in the FFT picture due to this sample’s very low Bi content. Nevertheless, it was possible to identify the Bi_2_O_3_ phase in the FFT analysis (Figure 2H), even though its intensity was very low. In some areas, it can be seen that Bi_2_O_3_ forms very small clusters, which may be responsible for the Bi_2_O_3_ phase reflections visible in the XRD (Figure 2F). Compared with the Bi_0.02_Co_2.98_O_4_ and Bi_0.2_Co_2.8_O_4_ samples, speckles from the Bi_2_O_3_ phase are visible on the FFT for Bi_0.2_Co_2.8_O_4_, while for sample Bi_0.02_Co_2.98_O_4_ these spots are not present.

**Figure 3:**
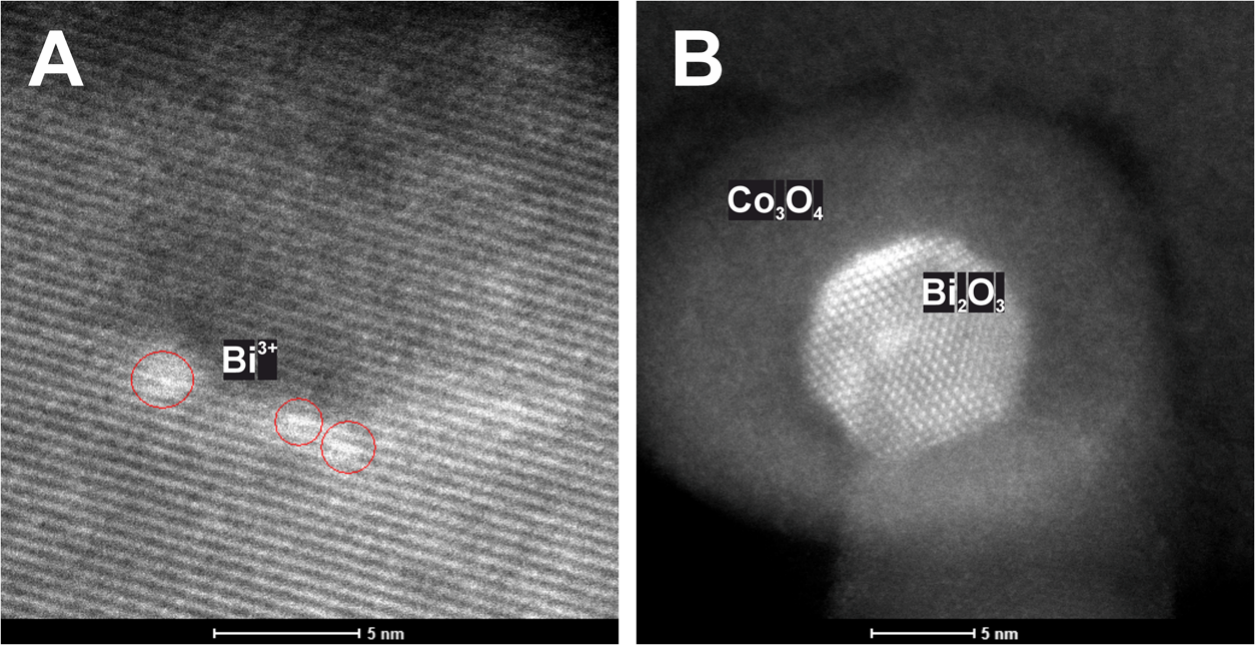
STEM images of the Bi_0.02_Co_2.98_O_4_ sample (A) and the Bi_0.2_Co_2.80_O_4_ sample (B). The locations of Bi^3+^ cations in the Bi_0.02_Co_2.98_O_4_ sample are marked in red. The segregation of bismuth into Bi_2_O_3_ crystallites in the Bi_0.2_Co_2.80_O_4_ sample is presented in the image on the right.

Further evidence of the atomic dispersion of bismuth (Bi) in the Co_3_O_4_ lattice was obtained from the analysis of the Bi_0.02_Co_2.98_O_4_ sample. A strong *Z*-contrast was achieved by employing a high-angle annular dark-field (HAADF) detector for scanning transmission electron microscopy (STEM) images. This allowed for a clear distinction between bismuth and cobalt, with the bismuth-containing areas appearing significantly brighter in the images. These bright spots, measuring approximately 1–1.5 Å in diameter (Figure 3A), are interpreted as individual Bi^3+^ cations or small clusters of a few atoms distributed across the cobalt spinel surface. Conversely, for the Bi_0.2_Co_2.8_O_4_ sample, Bi_2_O_3_ nanocrystals can be detected, Figure 3B. A thorough analysis of STEM imaging of the Bi_0.2_Co_2.8_O_4_ sample revealed that Bi-related bright spots cannot be detected in the Co_3_O_4_ fragments, indicating that Bi segregation hinders bismuth incorporation into the spinel lattice.

**Figure 4:**
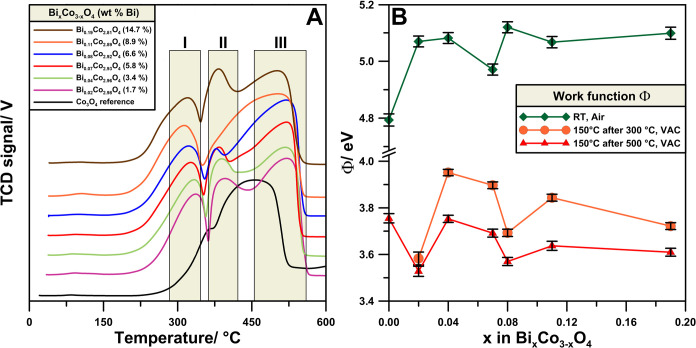
(A) H_2_-TPR reducibility results of Bi_*x*_Co_3–*x*_O_4_ samples. (B) Contact potential difference expressed as work function (Φ), measured in ambient atmosphere and under vacuum after annealing at 300 and 500 °C.

The redox properties of the Bi-doped Co_3_O_4_ catalysts can be successfully studied using temperature-programmed reduction with hydrogen (Figure 4A). The reducibility profile of undoped Co_3_O_4_ is typical for this material precipitated and calcined at high temperatures.^46^ The addition of Bi results in three distinct changes in the H_2_-TPR profile. First, an additional maximum appears above 400 °C due to the reduction of Bi from Bi_2_O_3_.^47^ Second, the low-temperature peak of Co^3+^ from the spinel lattice shifts toward lower temperatures. Third, the high-temperature tail of the reduction peak shifts toward higher temperatures. The low-temperature shift of the initiation of the reduction process (maximum I in Figure 4A) can be due to the destabilization of the spinel lattice and weakening of the metal–oxygen bonds by incorporating Bi. At lower temperatures, Bi^3+^ would segregate as Bi_2_O_3_ and undergo independent reduction indicated by the maximum II in Figure 3A. The remaining heterogeneous system appears to stabilize the remaining CoO, as observed by the shift in the maximum III in Figure 4A.

Changes in the surface electronic properties of the studied catalysts were followed by using the work function measurements with a Kelvin probe mounted in a vacuum chamber. It was found that although Bi doping increases the Φ of the clean surface (Figure 4B, after 300 and 500 °C in vacuum), the water-covered surface has a lower Φ (Figure 4B, RT, Air). The increase in the work function results in weaker adsorption of water, also evidenced by a shift toward lower temperatures of the water desorption curves for the Bi-doped samples (Figure S4). The most pronounced effect on the work function is observed for the Bi doping level 5.8 wt % (Bi_0.07_Co_2.93_O_4_), which is similar to the optimal amount for the best deN_2_O activity in dry and wet atmospheres (Figure S2, inset). Therefore, it can be concluded that a weaker interaction of water molecules with the spinel surface due to increased work function results in a higher activity of the water-covered surface.

The surface electronic properties of the Bi_*x*_Co_3–*x*_O_4_ materials are reflected in their charge transfer resistance (*R*_ct_^OH^) in the alkaline electrolyte, determined using a fitted equivalent circuit,^48^ where the inner sphere electron transfer in the reaction of water oxidation shows a pronounced minimum for the sample with the highest Φ of the clean surface and the lowest work function of the water-covered surface (Bi_0.07_Co_2.93_O_4_), as shown in Figure 4A (impedance spectra available in SI—Figure S5). Complementary electrochemical studies performed using the [Fe(CN)_6_]^3–^/[Fe(CN)_6_]^4–^ redox system show that for the outer sphere electron transfer reaction, *R*_ct_^Fe^, determined using Randles circuit (*R*_ct_^Fe^),^49^ reaches the value close to the registered minimum for the same composition as in the alkaline electrolyte. This indicates that further addition of Bi to Co_3_O_4_ does not increase the modification of its electronic properties, which can be justified by an increased Bi_2_O_3_ segregation indicated by the XRD patterns.

The [Fe(CN)_6_]^3–^/[Fe(CN)_6_]^4–^ redox system was also used to determine the electrochemical properties of the studied materials, such as the electrochemically active surface area (ECSA) and relative separation of the oxidation and reduction peaks (χ^0^) (Table 2). The cyclic voltammograms, impedance spectra, and detailed methods used to determine the parameters are presented in Supporting Information, Section S4 and Figures S6 and S7.

**Table 2:** Summary of Parameters Obtained for the Tested Catalyst Samples: *S*_BET_, *C*_dl_, ECSA, χ^0^; Overpotential of the OER Determined for the 10 mA cm^2^ Current (η) and Tafel Slope

sample (wt % Bi)	*S*_BET_ [m^2^ g^–1^]	*C*_dl_ [mF]	ECSA [mm^2^]	χ^0^	η [mV]	Tafel slope [mV dec^–1^]
Co_3_O_4_ (0%)	9	0.13	2.7	4.1	393	47
Bi_0.02_Co_2.98_O_4_ (1.7%)	35	0.19	3.3	2.8	387	48
Bi_0.04_Co_2.96_O_4_ (3.4%)	39	0.37	4.6	2.4	393	53
Bi_0.07_Co_2.93_O_4_ (5.8%)	46	0.53	4.9	1.7	350	48
Bi_0.08_Co_2.92_O_4_ (6.6%)	58	0.31	5.0	1.7	365	46
Bi_0.11_Co_2.89_O_4_ (8.9%)	79	1.05	5.2	1.6	361	75
Bi_0.19_Co_2.81_O_4_ (14.7%)	53	0.47	4.9	1.7	419	61

When Bi was added to the samples, significant ECSA and *S*_BET_ growths were observed. It resulted from earlier hydrolysis of Bi^3+^ ions forming nucleation centers for Co(OH)_2_. The changes in the surface area are further discussed along with the cobalt accessibility values determined with CV. The increase in activity of the bismuth-doped catalyst in the [Fe(CN)_6_]^3–^/[Fe(CN)_6_]^4–^ redox system is related to the increase in the surface area capable of carrying out the electrode reaction. Additionally, an inverted trend was observed in the relative separation of the Fe^3+/^Fe^2+^ signals. The diminished tendency of χ^0^ values is evidence of improving the electrode transfer kinetics rate because obtained values for higher Bi content are closer to the theoretical value of (χ^0^ = 1).^50^

Measurements of the double-layer capacitance (*C*_dl_) were performed in 0.1 mol L^–1^ KOH, and results are presented in Table 2 and SI Figure S8. The obtained trend is similar to that previously discussed in the [Fe(CN)_6_]^3–^/[Fe(CN)_6_]^4–^ redox system (ECSA and *S*_BET_).

**Figure 5:**
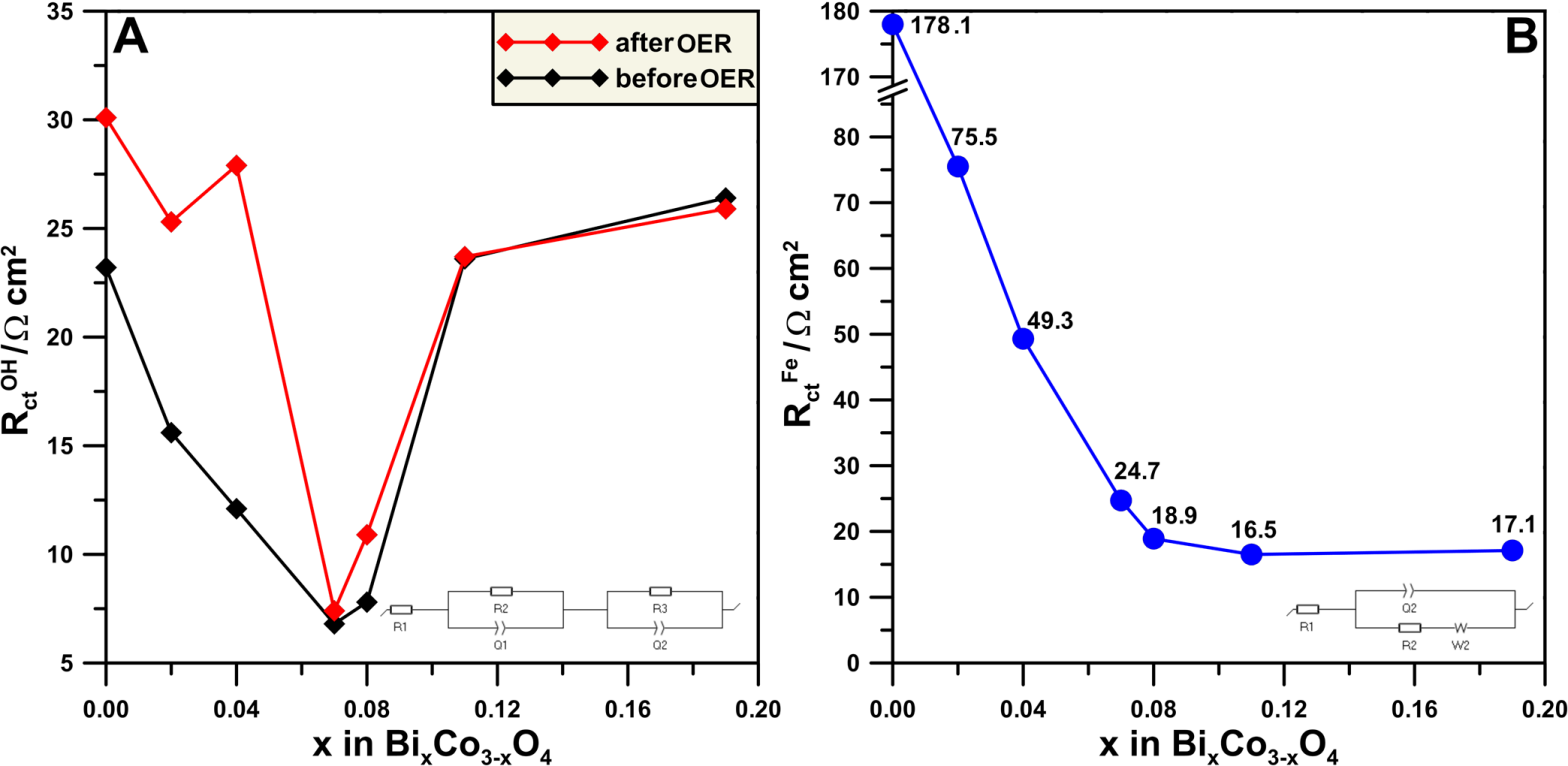
Charge transfer resistance values obtained in (A) 0.1 mol L^–1^ KOH, recorded before and after OER tests, for the samples with Bi_*x*_Co_3–*x*_O_4_ general formula, *x* = (0; 0.02; 0.04; 0.07; 0.08; 0.11; 0.19) denoted as *R*_ct_^OH^; (B) 5 mmol L^–1^ K_3_[Fe(CN)_6_]/K_4_[Fe(CN)_6_] redox system + 0.1 mol L^–1^ (*R*_ct_^Fe^).

**Figure 6:**
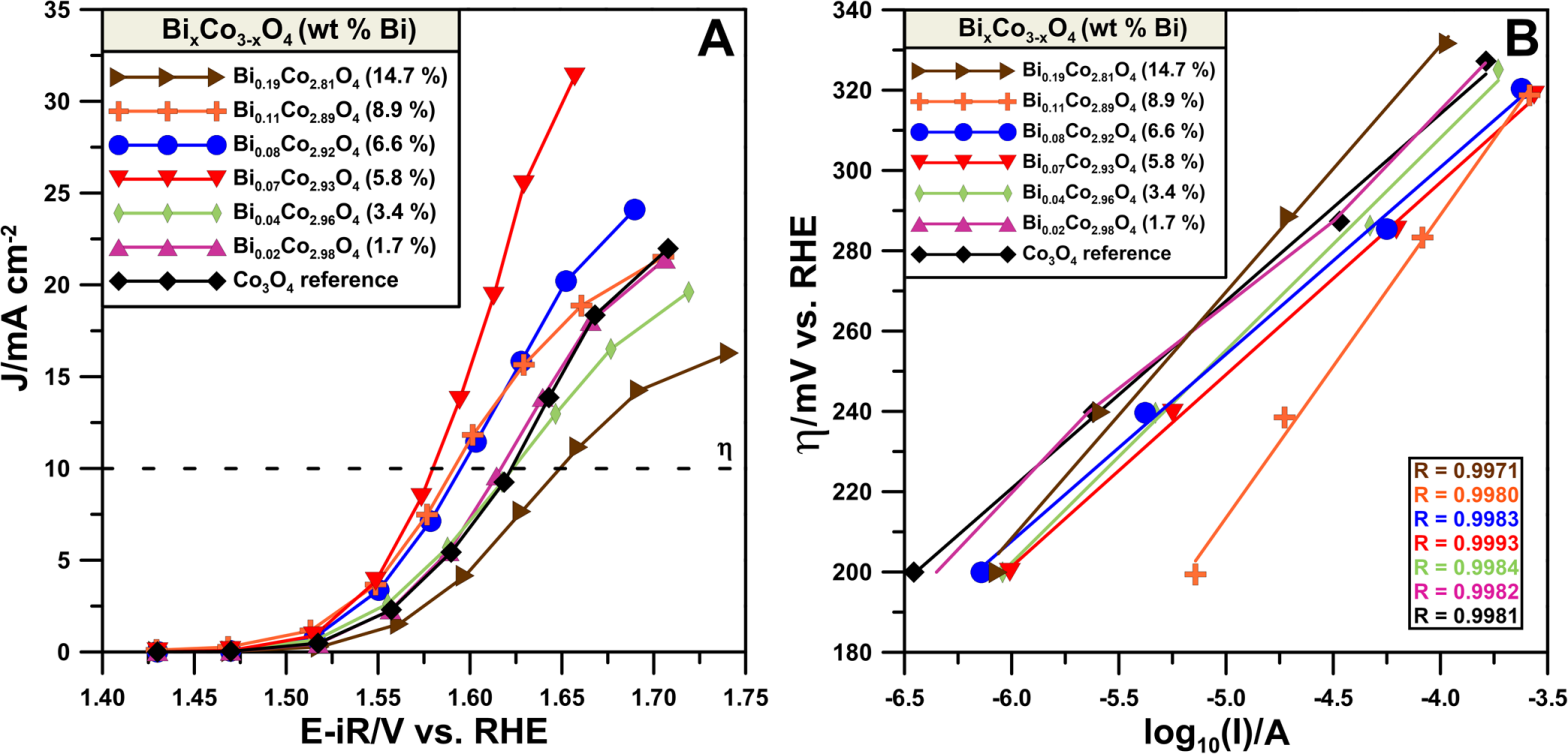
(A) Steady-state OER activity of the Bi_*x*_Co_3–*x*_O_4_ catalysts. (B) Tafel plots used for determining the Tafel slope.

The OER activity tests confirm the enhancement of catalytic properties upon Bi doping (Figures 6A and S9). Since the Bi_2_O_3_ has insulating properties,^51^ it does not contribute to the electrode reaction. Thus, segregated Bi_2_O_3_ should have a hindering effect on the OER activity due to a decrease in the electronic conductivity of the catalytic system. This effect is observed for the samples with the highest bismuth content in terms of both the water oxidation current (Figure 6A) and the *R*_ct_^OH^ in the alkaline environment, where the impedance studies were conducted before the OER (chronoamperometric activity studies and following LSV cycles for preliminary stability studies) and after the OER (Figure S9). Interestingly, when Bi is added above a certain threshold, which corresponds to the maximum OER activity, the stability of the catalysts increases, as indicated by the consistent value of *R*_ct_^OH^ before and after the OER (Figure 5A). Tafel plot analysis (Figure 6B and Table 2) indicates that extensive segregation of Bi_2_O_3_ for the highest Bi content samples results in a substantial deterioration in the reaction kinetics, suggesting a change in the reaction mechanism, possibly mass transfer limitations due to covering active centers by an inactive Bi_2_O_3_. The presence of segregated Bi_2_O_3_ suggests the formation of a heterojunction. The heterojunction that forms may contribute to activity in general. However, the experimental data show that for an increased Bi_2_O_3_ segregation, the OER activity decreases. Thus, the observed activity enhancement can be attributed to Bi modifying the Co_3_O_4_ lattice.

**Figure 7:**
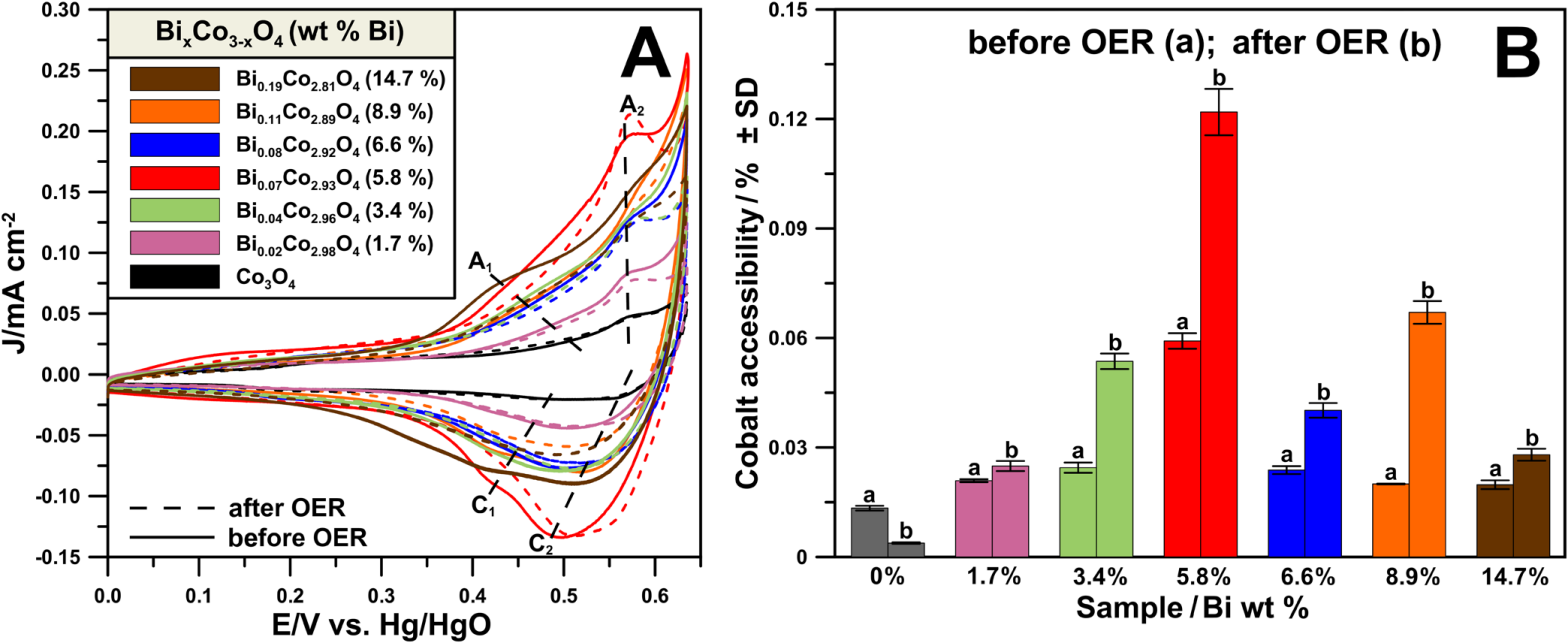
(A) CVs registered in the cobalt oxidation–reduction potential range in 0.1 mol L^–1^ of KOH before and after the onset of OER reactivity testing. (B) Cobalt accessibility values were obtained before and after the onset of the OER reactivity testing.

The measurements of Bi_*x*_Co_3–*x*_O_4_ catalysts (where *x* = 0; 0.02; 0.04; 0.07; 0.08; 0.11 and 0.19) were performed using CV for ν = 5 mV s^–1^ in 0.1 mol L^–1^ KOH before CA and after LSV measurements. The voltammograms are presented in Figure 7A. The oxidation peaks A_1_ and A_2_ in the potential range 0.4–0.56 V and the corresponding reduction signals C_1_ and C_2_ can be visible. The Co^2+^/Co^3+^ and Co^3+^/Co^4+^ redox transitions can explain the presence of the signals and relate to the occurrence of the reactions described by eqs 1 and 2(52−54)
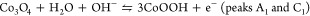
1
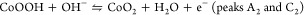
2The cobalt reducibility (accessibility, Figure 7B) follows the nonmonotonous trend of the OER reactivity and the work function changes. It increases for the low Bi levels, reaching a maximum value for the most active sample. The quantity of reducible cobalt decreases despite the high values of the specific surface areas and ECSA for higher Bi loadings (Figure S10A,B). The observed trend can be justified based on the initial incorporation of Bi into the spinel lattice, which increases the reducibility of cobalt ions in Co_3_O_4_. The segregation of Bi for higher doping results in the blocking of the spinel surface. Further, a limited Bi incorporation was evidenced by microscopic imaging, which may cause increased work function, decreased OER activity, and decreased redox activity of cobalt sites.

**Figure 8:**
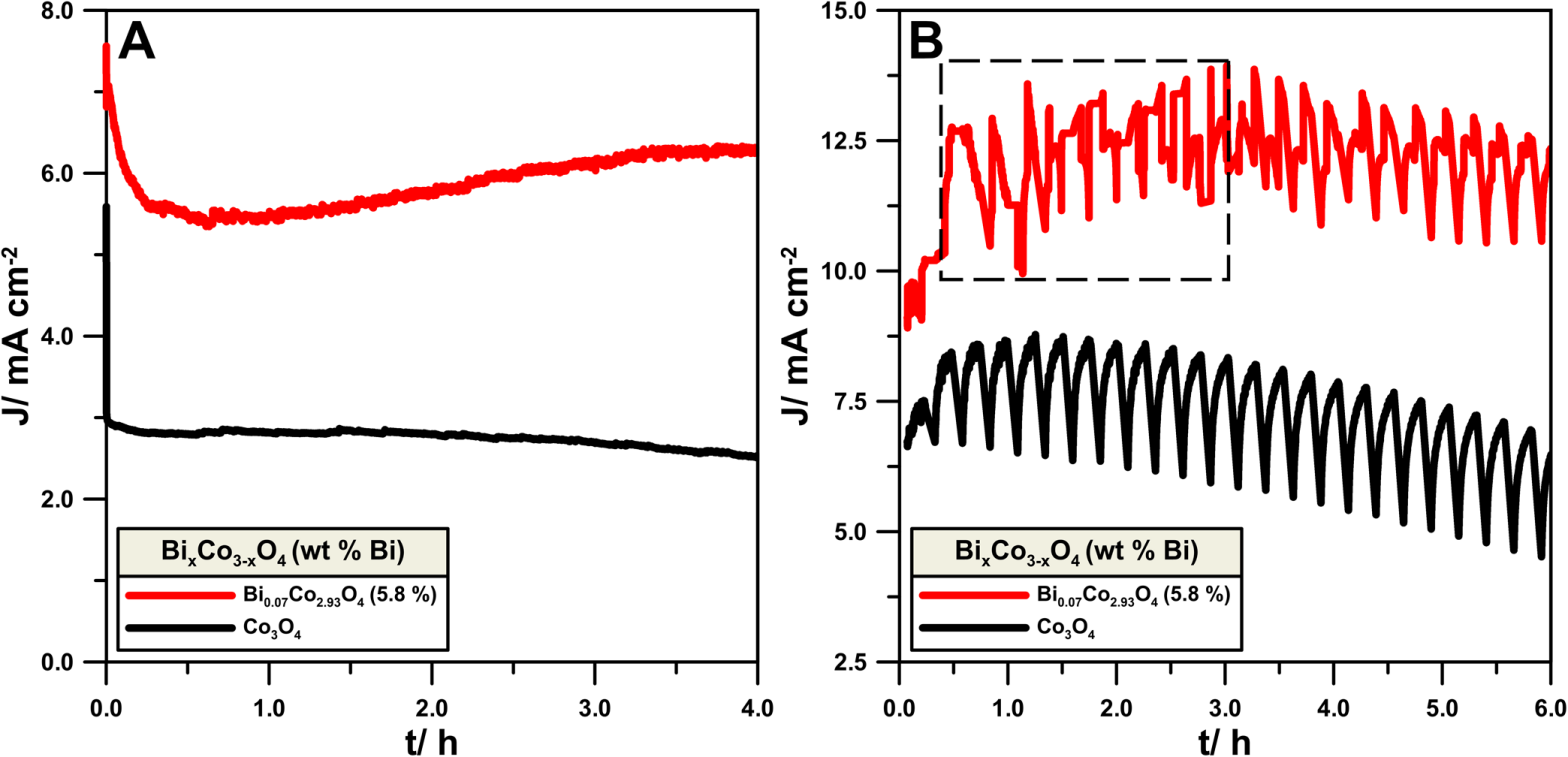
OER stability tests in (A) chronoamperometric conditions at 1.65 V vs RHE and (B) cycles of 10 min chronoamperometric steps at 1.7 V vs RHE.

The improved stability of the best Bi-doped Co_3_O_4_ compared to the reference Co_3_O_4_ is presented in Figure 8. The 4 h single CA stability test conducted at 1.65 V vs RHE, after the initial drop, revealed that Co_3_O_4_ starts to lose the initial activity after 2 h, while the Bi-doped sample provides a higher anodic current, which slightly increases over time. That is evidence of catalyst activation during the time (Figure 8A). Additional stability investigations were performed during a 6 h test at 1.7 V vs RHE, with cyclic steps of a constant potential (Figure 8B). The improved activity and stability of Bi-doped Co_3_O_4_ were confirmed.

DFT studies were conducted to explain the experimental results and elucidate the mechanism behind improving the OER activity by doping cobalt spinel with bismuth. Technical background such as the assumption used for calculations of bulk Co_3_O_4_ and Bi–Co_3_O_4_ structures, slab models of Co_3_O_4_ and Bi–Co_3_O_4_ (100) surfaces, OER free energy diagrams principles, information about the calculation of potential-determining step of OER process as well as OER intermediates adsorption DFT studies model, and projected electronic density of states (DOS) can be found in Figures S11–S15 and the accompanying description in the Supporting Information, Section S5.

**Figure 9:**
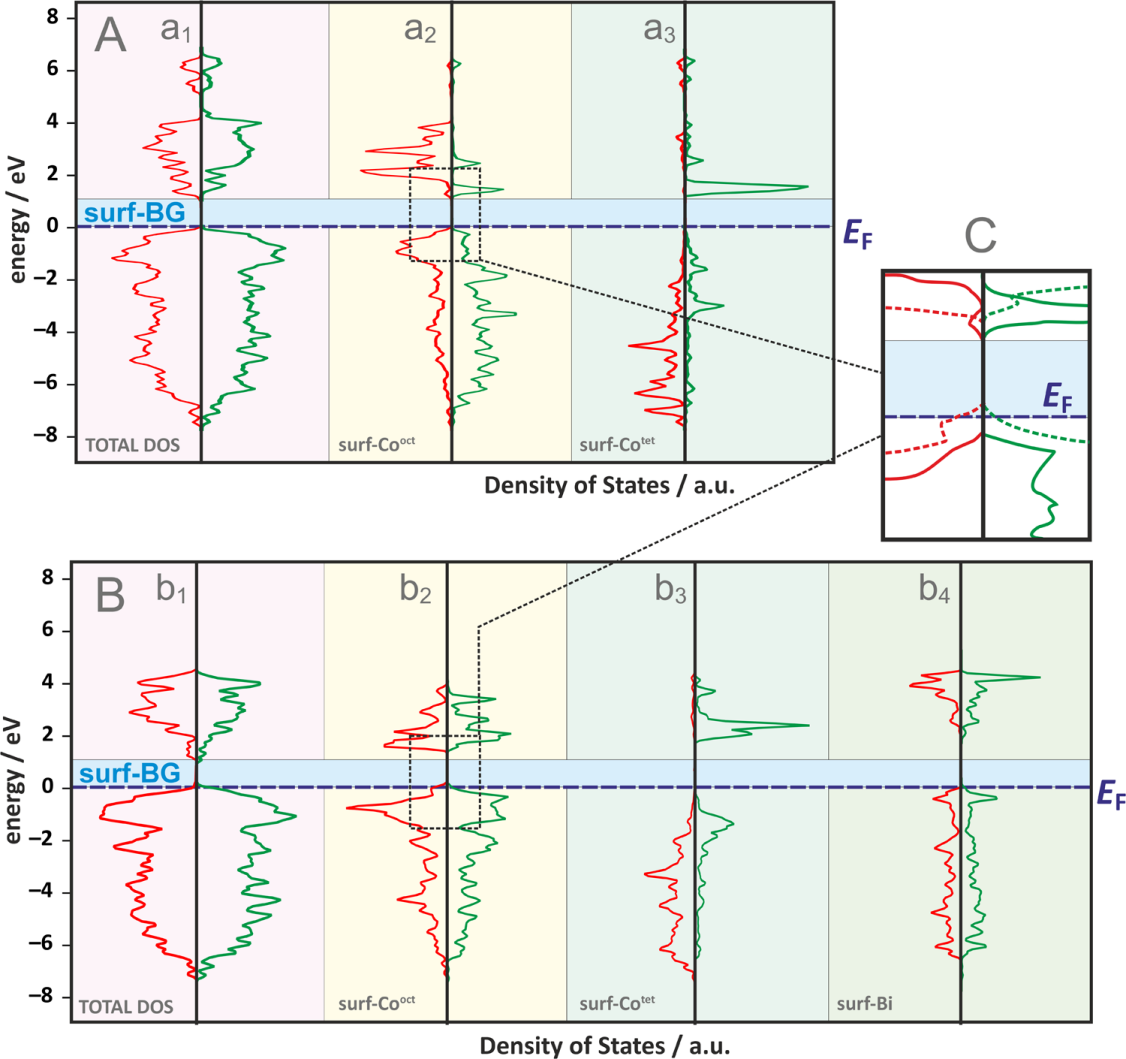
Total and partial electronic density of states plots for (A) bare (a_1_–a_3_) and (B) Bi-doped (b_1_–b_4_) (100) surfaces of cobalt spinel together with the magnified region of Co_(surf)_^3+^ pDOS near the *E*_F_ level for both catalysts (bare spinel; solid lines; Bi-doped spinel: dashed lines). (C) The majority (α) and minority (β) spin states are marked in red and green, respectively.

The comprehensive density of state (DOS) plot for the slab model of the (100) surface of undoped cobalt spinel is shown in Figure 9. Spin symmetry remains high, but there is a clear reduction in the band gap value compared to the bulk material (from 1.7 to ∼1.0 eV, see Figure S15). Such an effect is typical for surfaces and results from the presence of a significant number of broken bonds. Figure 9a_2_,a_3_ depicts the partial DOS plots for surface Co^oct^ and Co^tet^ cations that may participate in the OER process by stabilizing intermediate products. The octahedral cation on the (100) surface, due to the reduction in coordination from 6 to 5 (and consequently a symmetry change from *O_h_* to *C*_4*v*_), acquires a magnetic moment, corresponding to two unpaired electrons (*S* = 1, (d*_xz_*,d*_yz_*)^4^(d_*xy*_)^1^(d_*z*2_)^1^ configuration). The tetrahedral cation, in this case with a bridging geometry and double coordination, retains a high-spin state (*S* = 3/2, in line with the (d_*z*2_,d_*z*2_–_*y*2_)^4^(d_*xy*_)^1^(d_*xz*_)^1^(d_*yz*_)^1^ electronic configuration resulting from the local *C*_2*v*_ symmetry).

Doping cobalt spinel with bismuth in octahedral positions does not significantly affect the overall DOS plot (Figure 9b_1_), which is associated with the isoelectronic nature of the substituted cations (Bi^3+^ → Co^3+^). Bismuth incorporated into the lattice is characterized by a large local band gap (∼2 eV) and zero spin polarization. Its presence does not significantly impact the partial DOS of surface tetrahedral cobalt (compare Figure 9a_3_,b_3_), but it noticeably increases the 3d density of states for surface octahedral cations. This effect is illustrated in Figure 9C, which shows a magnified region of the partial DOS plot for the Co_(surf)_^3+^ ion in the pure catalyst (solid lines) and the Bi-doped catalyst (dashed lines) near the Fermi level. A clear shift in the conduction band edge is visible, resulting from a partial electron density transfer to the cobalt cation. This increased density of states near the Fermi level may explain the improved OER activity of the Bi_*x*_Co_3–*x*_O_4_ system through a better-balanced distribution of potential steps, as illustrated in the free energy diagram below.

**Figure 10:**
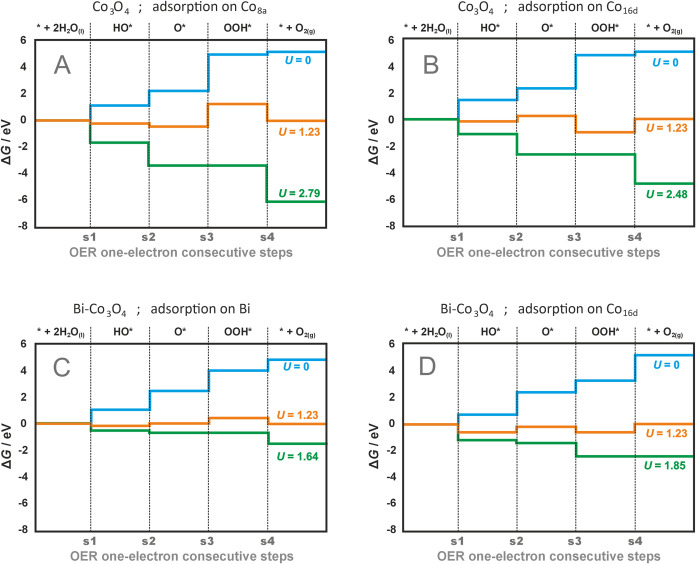
Standard free energy diagram for the OER at zero potential (*U* = 0, blue line), equilibrium potential for oxygen evolution (*U* = 1.23, orange line), and at the potential for which all steps become downward (green line) at pH = 0 and *T* = 298 K over the (A) Co_8a_ and (B) Co_16d_ cations of pure Co_3_O_4_ as well as the (C) Bi_16d_ and (D) Co_16d_ sites of Bi-doped cobalt spinel.

The DFT-optimized geometries of the OER intermediates bound to different active sites of the Co_3_O_4_ and Bi_*x*_Co_3–*x*_O_4_ catalysts are presented in Figure S14, whereas the corresponding binding energies are summarized in Table 3. For modeling, we selected the most important sites occurring in pure (Figure S14a_1_–b_3_) and doped bismuth cobalt spinel (Figure S14c_1_–d_3_). The differences in the binding energy of the individual intermediates in the OER process to the active centers (Table 3) result from varying orbital overlaps and the energy matching of the oxygen atom p orbitals with the d bands of the cations acting as adsorption centers. These binding energies were used to construct a standard free energy diagram of the OER process (Figure 10) to thoroughly understand the structure–performance relationship of the Co_3_O_4_ and Bi_*x*_Co_3–*x*_O_4_ catalysts.

**Table 3:** Binding Energies of HOO*, HO*, and O* Adspecies Calculated for Different Active Sites of Investigated Parent and Bi-Doped Cobalt Spinel Catalyst

	binding energy		
adsorption site			
Co_8a_ site on Co_3_O_4_	0.731	2.168	4.840
Co_16d_ site on Co_3_O_4_	1.015	2.251	4.618
Bi_16d_ site on Bi_*x*_Co_3–*x*_O_4_	0.769	2.561	4.123
Co_16d_ site on Bi_*x*_Co_3–*x*_O_4_	0.983	2.301	3.856

The OER activity of the octahedral Co^3+^ in the 16d position is higher than the tetrahedral Co^2+^ in the 8a position (Figure 10a,b), which agrees with the reported relationship, both experimental and theoretical.^55^ Nevertheless, the calculated potential for which all steps become downward is relatively high, equal to 2.48 and 2.79 V for Co^3+^ and Co^2+^ respectively. Introducing Bi^3+^ into the octahedral sites resulted in the creation of a new active center and modification of the Co_16d_^3+^ center in the spinel lattice. Both centers show improvement in the OER activity by decreasing the potential of the oxidation of O* to HOO* (eq S3, SI). However, in the case of adjacent Co_16d_^3+^, the influence of bismuth is so pronounced that the potential-determining step changes from the oxidation of O* to the last step—oxidation of HOO* and release of the oxygen molecule (eq S4). Both centers exhibit substantially decreased calculated potential for which all steps become downward, equal to 1.64 and 1.85 V for Bi_16d_^3+^ and Co_16d_^3+^, respectively. Electron transfer between the electrocatalyst and the oxygen intermediates is significantly related to the electronic states around the Fermi level.^56^ Computational calculations show that Bi doping of the Co_3_O_4_ surface significantly increases its density of states just below the Fermi level (Figure 9C). The calculated electronic changes in the Co_3_O_4_ structure are reflected in the experimentally derived charge transfer resistance of the inner sphere electron transfer in the oxygen evolution reaction and, thus, the enhanced reaction kinetics.

## 4 Conclusions

For the first time, cobalt spinel doped with bismuth was investigated as a catalyst for the oxygen evolution reaction, achieving promising activity (350 mV@10 mA cm^–2^) by reducing the value of the OER overpotential by 43 mV compared to the bare Co_3_O_4_. Incorporating Bi atoms into the Co_3_O_4_ lattice also improved long-term stability compared to an undoped sample in a 4-h single CA scan provided for the potential of 1.65 V vs RHE as well as a 6-h cyclic test examined by a chronoamperometry measurement at 1.7 V vs RHE. Theoretical calculations revealed that atomically dispersed Bi dopants are more likely to replace octahedral Co^3+^ than tetrahedral Co^2+^. An increased density of states below the Fermi level modifies the electronic structure of the Bi-doped spinel. Experimentally, Bi doping weakens the Co–O bond, improving the material’s reducibility. Furthermore, the work function of the Bi-doped Co_3_O_4_ increases, leading to a weaker interaction with water molecules, which may facilitate the interaction of the active centers with the adsorbates. The substituted Bi is an active site in OER processes, enhancing oxygen desorption. At the same time, the activity of the neighboring Co atoms is also substantially enhanced. This improvement leads to an increased intrinsic activity of Bi-doped Co_3_O_4_.

## Supporting Information Available

Research scheme; experimental details; N_2_O decomposition studies results; additional microscopic images; desorption profiles recorded during the work function measurements; additional electrochemical characterization results; details and background on the density functional theory calculations (PDF)
