# Supplementary material for: Modulation of the Electronic Properties of Co3O4 through Bi Octahedral Doping for Enhanced Activity in the Oxygen Evolution Reaction
Source: ACS Catal. 2025 Mar 6;15(6):4746–58. doi: 10.1021/acscatal.4c07911 (PMC11934081; doi:10.1021/acscatal.4c07911)
Supplement: Supplementary file 1 — cs4c07911_si_001.pdf [file cs4c07911_si_001.pdf]

Supporting Information
for

Modulation of the Electronic Properties of Co_3O_4 through Bi Octahedral Doping for Enhanced Activity in the Oxygen Evolution Reaction

Damian Gorylewski[a], Filip Zasada[b], Grzegorz Słowik[a], Magdalena Lofek[b], Gabriela Grzybek[b], Katarzyna Tyszczuk-Rotko[a], Andrzej Kotarba[b], Paweł Stelmachowski[b]*

[a] Maria Curie-Sklodowska University, Faculty of Chemistry, Institute of Chemical Sciences, Department of Analytical Chemistry, Maria Curie-Sklodowska Sq. 3, 20-031 Lublin, Poland

[b] Jagiellonian University, Faculty of Chemistry, Gronostajowa 2, 30-387 Krakow, Poland

* Corresponding author: e-mail address: pawel.stelmachowski@uj.edu.pl

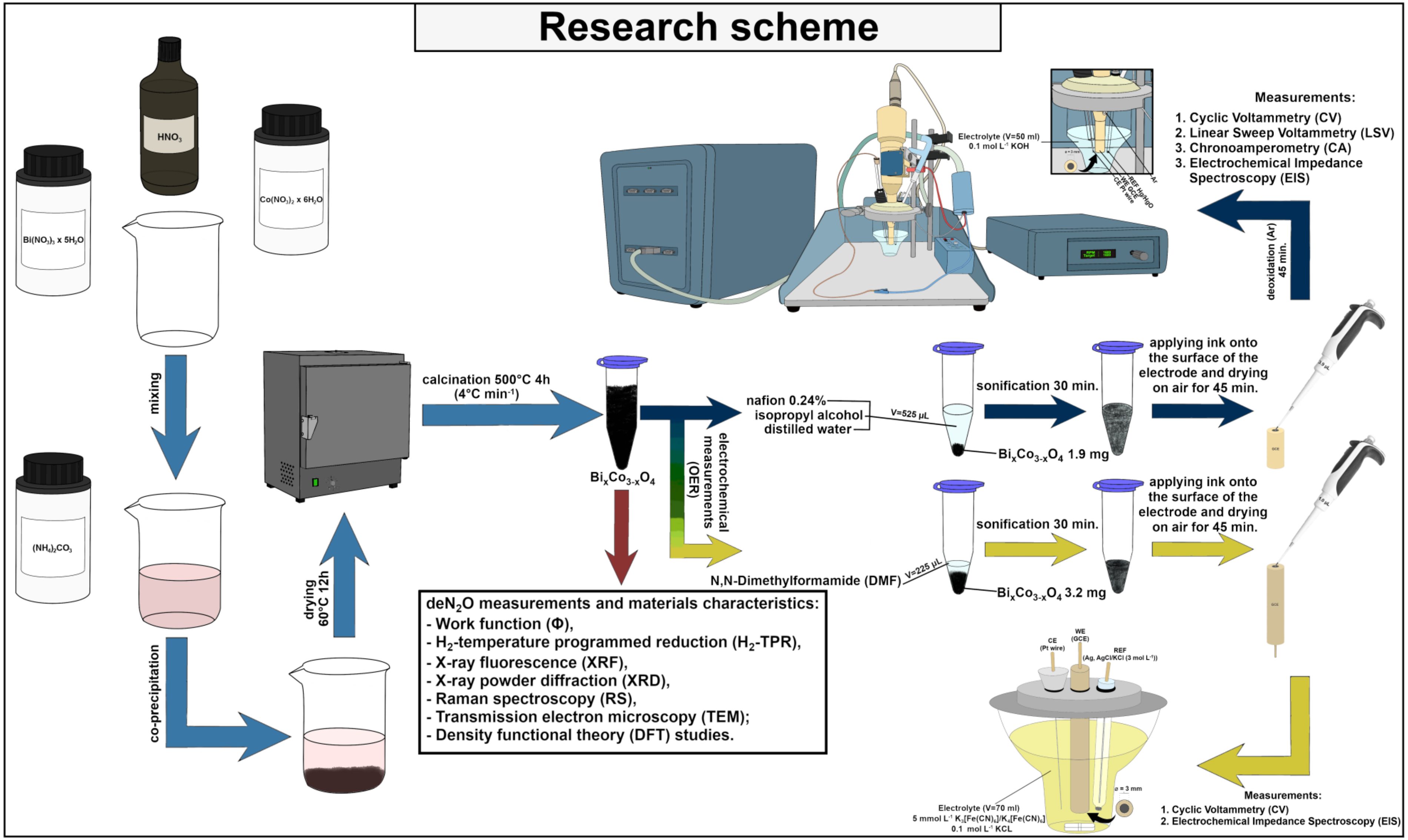

Figure S1. Research scheme.

Section 1 – Experimental

1.1 Synthesis details

Table S1. The masses of $Bi(NO_3)_3 \times 5H_2O$ and $Co(NO_3)_2 \times 6H_2O$ dissolved in 2 mol L^{-1} HNO_3 aqueous solution in order to obtain 1 mol L^{-1} concentration of cations.

**Theoretical formula ($Bi_xCo_{3-x}O_4$)**	**Mass of $Bi(NO_3)_3 \times 5H_2O$ (g)**	**Mass of $Co(NO_3)_2 \times 6H_2O$ (g)**
Co_3O_4	0.00	4.37
$Bi_{0.02}Co_{2.98}O_4$	0.05	4.34
$Bi_{0.04}Co_{2,96}O_4$	0.10	4.31
$Bi_{0.07}Co_{2.93}O_4$	0.17	4.26
$Bi_{0.08}Co_{2.92}O_4$	0.19	4.25
$Bi_{0.11}Co_{2.89}O_4$	0.27	4.21
$Bi_{0.19}Co_{2.81}O_4$	0.46	4.09

1.2 Physicochemical characterisation

1.2.1 XRF, XRD, RS and S_{BET} studies

Catalysts in the form of tablets with a diameter of 10 mm were used for analysis using X-ray fluorescence spectroscopy (XRF) measurements conducted on Thermo Scientific ARL QUANT'X apparatus controlled by UniQuant software, using 1 mm collimator and Mylar Chemplex Industries, INC. foil. A rhodium anode was used as an X-ray source. The instrument was recalibrated with a series of metallic standards before measurements. X-ray diffraction analysis (XRD) was performed on a Rigaku MiniFlex diffractometer with a copper source and a range of 2 angle values from 15° to 90° with a 0.02° step. Transmission electron microscopy (TEM) imaging was conducted using a 200-mesh copper grid with deposited catalysts samples were inserted into a single-tilt holder and placed in the Titan G2 60−300 kV (FEI Company) electron microscope equipped with field emission gun, monochromator, three condenser lenses system, an objective lens system, an image correction (CS-corrector), Tecnai electron microscope, high-angle annular dark field detector (HAADF) and energy dispersive X-ray spectrometer (EDAX). All measurements were performed with an electron beam accelerating voltage of 300 kV. Raman spectroscopy (RS) experiments were performed on a Renishaw InVia spectrometer coupled with a Leica DMLM confocal microscope and a CCD detector. To record Raman spectra in the range from 100 cm^{-1} to 900 cm^{-1}, a laser with a wavelength of 785 nm was used. Experiments were performed at room temperature. To enhance the signal-to-noise ratio (S/N), 12 subsequent scans were accumulated. The samples' specific surface area (SSA) was investigated via nitrogen absorption-desorption measurements using Micromeritics ASAP 2010

instrument. Catalyst samples were outgassed at 623 K for 14 h under vacuum before the measurements were performed at 77 K. Raw data were analysed using the Brunauer-Emmett-Teller (BET) isotherm method. The reducibility of catalyst samples was examined using hydrogen temperature programmed reduction studies (H_2-TPR). Experiments were performed by placing 20 mg of the sample in a fixed-bed quartz flow microreactor system with a thermal conductivity detector (TCD3, Valco, Houston, TX, USA). A reducing gas mixture contained 5 % H_2 in Ar (99.999% both) was passed through the system with a 10 ml min^{-1} flow rate in the temperature range of 40 – 600 °C (10 °C min^{-1}). A cold trap was used to remove water during experiments.

1.2.2 Work Function Measurements (Φ)

The electronic properties of cobalt spinel catalysts doped with bismuth have been investigated by determination of work function (Φ). Measurements were carried out on the KP 6500 probe by McAllister Technical Services (Coeur d'Alene, ID, USA) utilising the Kelvin dynamic condenser method. A stainless-steel plate (3 mm diameter) with a determined work function (Φ = 4.3 eV) has been used as a reference electrode. Work function experiments were performed at room temperature on air and with the heating program under vacuum (base pressure below 5×10^{-9} mbar). In the vacuum, measurements were performed at 150 °C after heating to 300 °C and 500 °C. The obtained results are the average of 30 single measurement readings performed for each catalyst. Experiments were performed with the following parameters: vibration frequency = 120 Hz and amplitude = 40 a.u.

1.3 Electrochemical Measurements

1.3.1 OER Activity and Stability Studies

Electrochemical measurements were performed in a 50 ml electrochemical cell filled with 0.1 mol L^{-1} KOH were conducted on a Biologic BP-300 bi-potentiostat connected to a Biologic RC-10K rotator and controlled by EC-Lab® software. The electrolyte solution was deoxidised for 45 minutes with Ar (99.999%) before measurements. Moreover, the electrochemical cell was argon flushed during experiments. A rotating disc glassy carbon electrode (RDGCE; Als, Japan) with a diameter of 3 mm and rotation speed of 1600 rpm was utilised as a working electrode for all experiments. Furthermore, Hg/HgO (1 mol L^{-1} NaOH) served as the auxiliary electrode and platinum wire as a counter electrode. Before a series of measurements, RDGCE was polished on a polishing pad with Al_2O_3 suspension (grain diameter: 0.05 μm) and substantially purified by sonification in deionised water. Then, the prepared electrode was left to dry and was ready for ink drop casting.

Ink preparation was conducted by mixing 1.9 mg of finely powdered catalyst, $Bi_xCo_{3-x}O_4$, 0.375 mL of distilled water, 0.125 mL of isopropanol (EUROCHEM) as well as 25 μL of Nafion 5% solution (Sigma-Aldrich) and subsequently sonicated for 30 min. In the next step, 3.9 uL of the previously homogenised ink suspension was applied onto the pretreated RDGCE surface and air-dried for 45 minutes at 200 rpm. After this time, the sensor was ready to study. The ink preparation procedure and the specific ink volume applied onto the working electrode surface allowed us to obtain a 200 μg cm^{-2} catalyst loading level. For the RDGCE with a diameter of 3 mm and a geometric surface area of 0.07065 cm^2, 0.014 mg of catalyst was present on its surface. The applied potentials were recalculated to the reference hydrogen electrode's (RHE) potentials [1].

The experiment procedure started with the stabilisation of catalyst film deposited onto RDGCE. For this purpose, CV (Cyclic Voltammetry) scans at a potential range of 0.2−0.9 V vs. RHE with 10 cycles of scan rate (ν) of 100 mV s^{-1} and 20 mV s^{-1}, and subsequently, 5 cycles of ν = 10 mV s^{-1} were performed. Next, the series of CV scans at a potential range of 1.2 − 1.3 V vs. RHE, with the = 2 - 12 mV s−1 (Figure S8A), was conducted to determine double-layer capacitance (C_{dl}) (Figure S8B). Catalysts' electrochemical properties were investigated through CA (Chronoamperometry) OER-activity and LSV (Linear Sweep Voltammetry) OER-stability experiments. Chronoamperometric measurements were performed in nine 15-minute steps with potential changes from 1.43 V to 1.83 V vs RHE with a 50 mV step. The ohmic drop (IR) correction was done using a high-frequency impedance measurement (EC-Lab® software - ZIR technique) and an averaging of uncompensated resistance registered before and after CA experiments. Liner sweep voltammograms were registered from 0 V to 2 V vs RHE ten times for ν = 5 mV s^{-1}. In LSV studies, IR correction was conducted by averaging PEIS (Potentiostatic Electrochemical Impedance Spectroscopy) measurements recorded at frequencies (f) = 100 kHz-100 mHz at 1.55 V vs RHE before and after the LSV stability experiment. These PEIS measurements were also utilised to determine charge transfer resistance (R_{ct}^{OH}) factors and their changes before and after OER experiments.

Additionally, CV measurements in the potential range from 0 V to 0.635 V vs. Hg/HgO for ν = 5 mV s^{-1} were carried out for each sample before CA and LSV activity tests (before OER) and after those experiments (after OER) to examine the oxidation (A_1 and A_2) and corresponding reduction (C_1 and C_2) peaks originating from Co^{2+}/Co^{3+} and Co^{3+}/Co^{4+} redox transformations [2]. The oxidation A_2 peak area was calculated for selected $Bi_xCo_{3-x}O_4$ catalysts (where x = 0; 0.02; 0.04; 0.07; 0.08; 0.11 and 0.19) using Fityk v.1.3.1 software, a simple 2-

point baseline correction and Gaussian function fitting. The obtained results with unit [A V] were recalculated to the corresponding cobalt molar masses based on II Faraday's Law. The obtained values [mol] were recalculated to [mg] and divided by the previously explained catalyst loading factor (0.014 mg) and presented as a percentage. This parameter was defined as "cobalt accessibility".

Long-term stability tests were conducted via a separate series of chronoamperometric measurements. Before the CA, catalyst film deposited onto RDGCE was stabilised, as mentioned before, with CV scans. A 4 h single CA measurement was performed, with an applied potential of 1.65 V vs. RHE. Furthermore, chronoamperograms were recorded for 36 cycles (steps), 10 minutes each, with a total time of 6 h, and an applied potential of 1.7 V vs. RHE.

1.3.2 Physicochemical Properties of Catalysts Determined via Electrochemical Methods

Biologic SP-50E potentiostat controlled by EC-Lab® software was utilised for PEIS and CV measurements. Experiments were conducted in a 70 mL electrochemical cell filled with 5 mmol L^{-1} $K_3[Fe(CN)]_6/K_4[Fe(CN)]_6$ redox system and 0.1 mmol L^{-1} KCl (Supelco). The non-deoxygenated solution was stirred for 5 seconds before each measurement. A glassy carbon electrode (GCE; ALS, Japan) with a diameter of 3 mm was utilised as a working electrode for all experiments. Furthermore, Ag/AgCl (3 mol L^{-1} KCl) served as the auxiliary electrode and platinum wire as a counter electrode. Before a series of measurements, GCE was polished on silicon carbide paper (#2500, Buehler, Skovlunde, Denmark) and subsequently on alumina particle suspensions (0.05 μm) on a polishing pad. The sensor was washed in an ultrasonic bath filled with distilled water for 3 minutes after each step. The electrode was left to dry on air and ready for ink drop casting.

To obtain a 200 μg cm^{-2} catalyst loading level, an ink suspension consisting of 1.9 mg of fine powdered catalyst sample and 225 μL of dimethylformamide (DMF) was sonicated for 30 minutes. Next, 1.0 μL of ink was drop-casted onto a dried and polished GCE surface and left to dry for 45 minutes, with the sensor rotating at 200 rpm simultaneously.

The experiment procedure for every catalyst sample started with PEIS measurements recorded at frequencies (*f*) = 50 kHz-1 Hz at 0.2 V vs. Ag/AgCl (3 mol L^{-1} KCl). Next, CV scans were registered with an increasing scan rate from 5 to 500 mV s^{-1}.

Section 2 – deN_2O studies

Catalysts deN_2O activity was investigated via temperature-programmed surface reaction (TPSR) mode (Figure S2). Samples were fine powdered and sieved. Fractions of 0.2-0.3 mm (0.3 mg) were placed onto sintered glass in a quartz flow reactor. Measurements were performed in a dry and wet atmosphere, with a model gas (5% of N_2O in He) with 30 mL s^{-1} flow rate and estimated gas space velocity equal 7000 h^{-1}. During measurements in the presence of H_2O, apparatus was saturated with water vapor before measurements and model gas was additionally humidified.

At the beginning of each experiment, catalysts samples were temperature cleansed until 500 °C of reaction gases flow was reached. Individual components (H_2O, N_2, O_2 and N_2O) concentration levels were measured at quadrupole mass spectrometer RGA200, SRS (Stanford Research Systems, Sunnyvale, CA, USA). The decomposition of nitrous oxide was studied from 100 °C to 600 °C with a temperature increase of 10 °C min^{-1}.

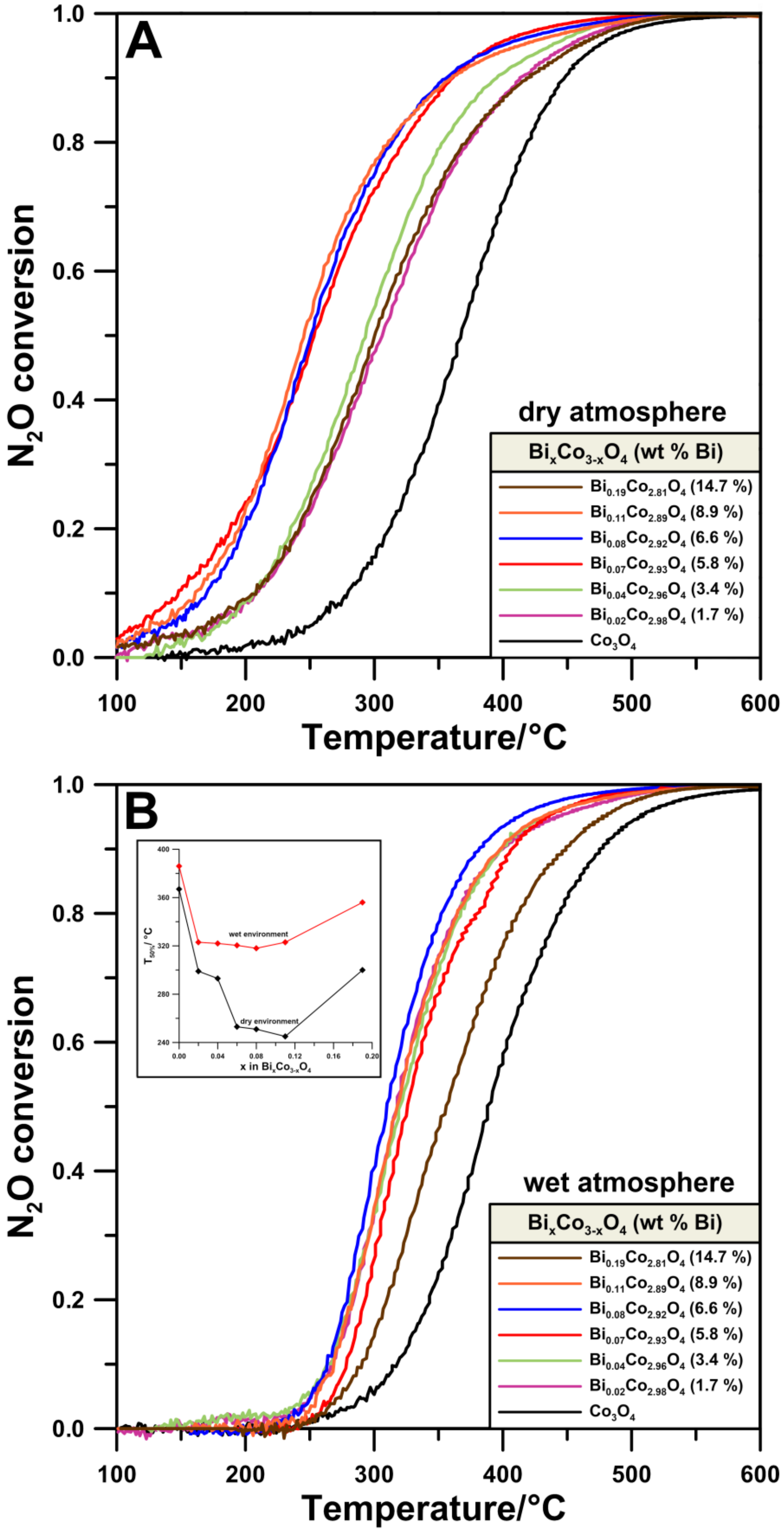

Figure S2. Comparison of deN_2O reaction conversion curves in a dry (A) and wet (B) environment, in terms of the bismuth subsidy effect for catalysts with the $Bi_xCo_{3-x}O_4$ general formula.

Section 3 – Physicochemical characteristics of catalysts

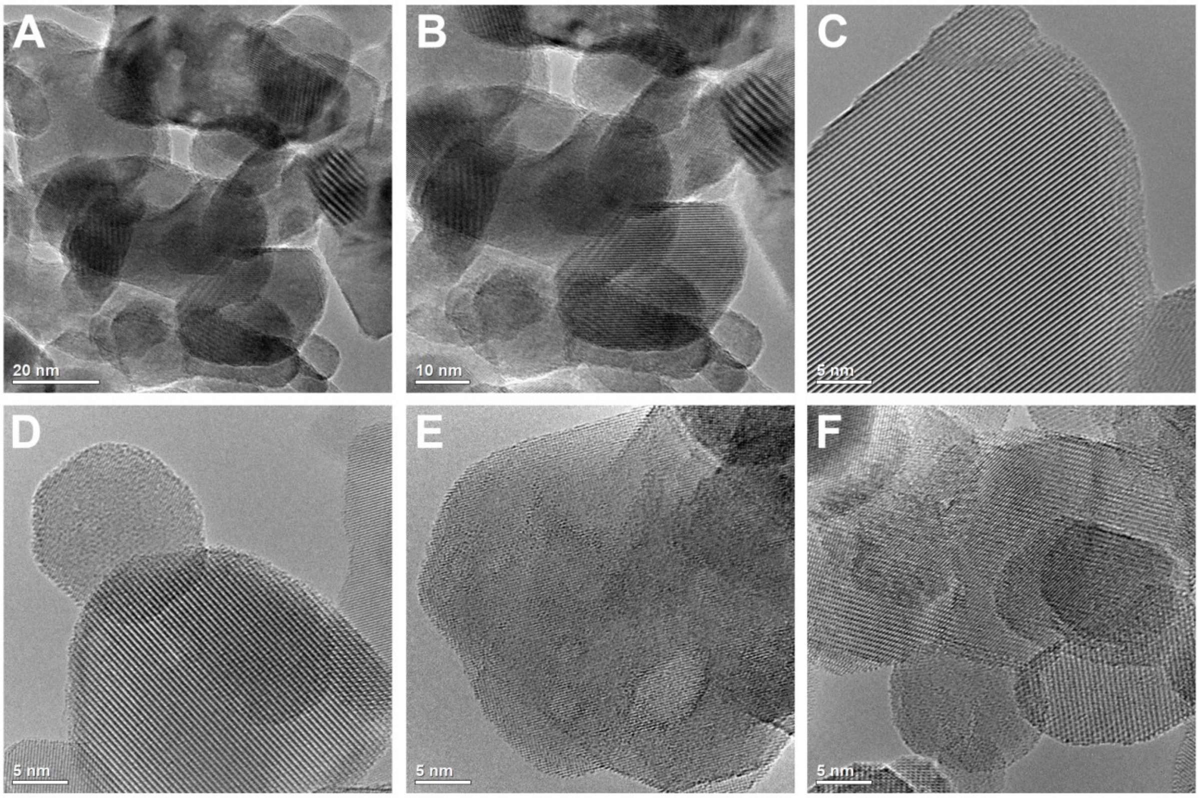

Figure S3. HR-TEM images of $Bi_{0.07}Co_{2.93}O_4$ sample.

The work function measurements were performed in ambient atmosphere and under vacuum. In vacuum the sample was heated first to 300°C, subsequently cooled down to 150°C and the measurement was carried out. Next, the samples were heated to 500°C, cooled down to 150°C, and the following measurement was carried out. During each step of heating the gasses desorbing from the catalysts’ surface were followed by a residual gas analyzer, quadrupole mass spectrometer. The desorption profiles are presented in Figure S4.

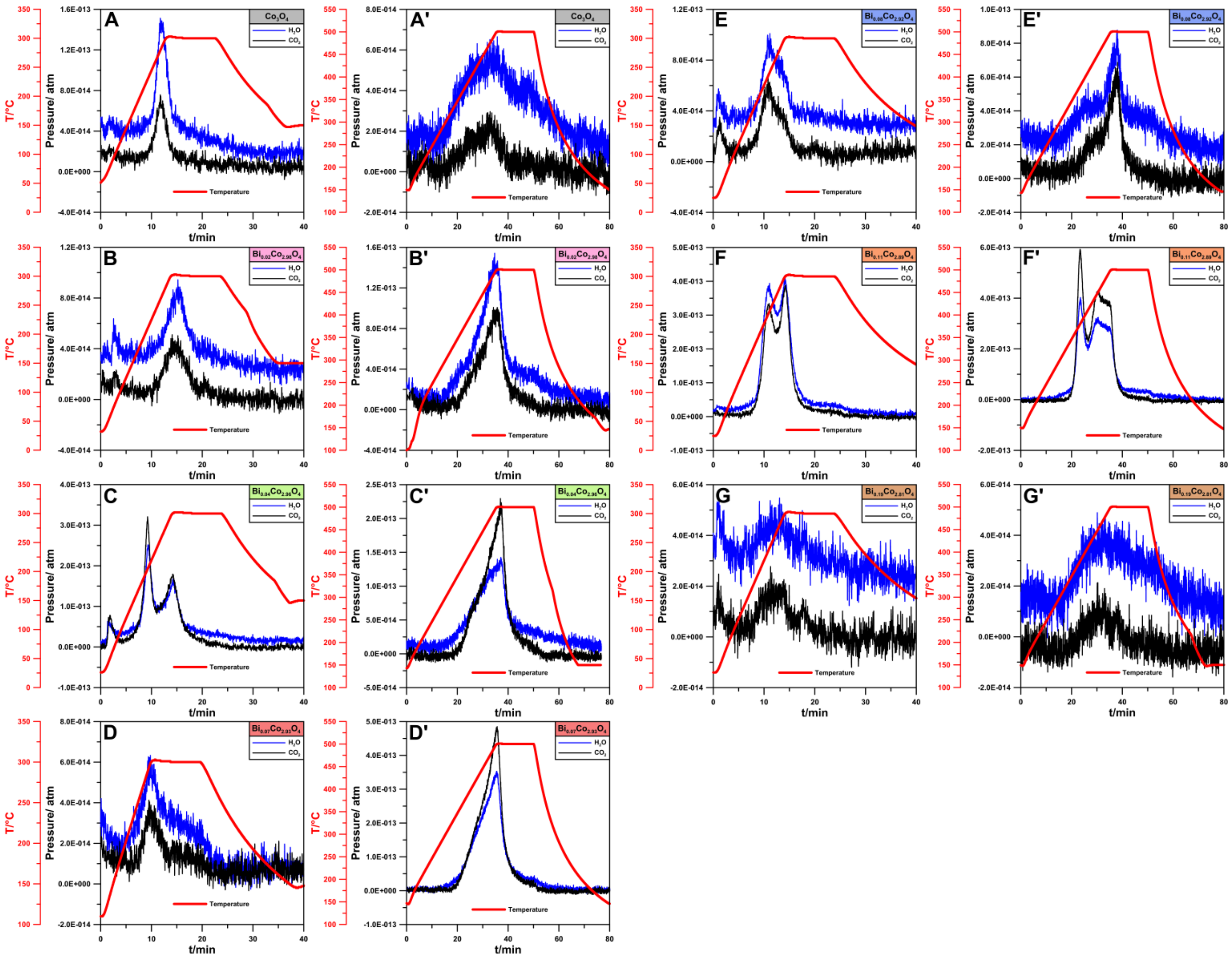

Figure S4. Desorption profiles recorded during the work function measurements in vacuum.

Section 4 – Electrochemical Measurements

In order to determine charge transfer resistance (R_{ct}^{Fe}), ECSA (Electrochemically Active Surface Area) and relative separation of the oxidation and reduction peaks (χ^0), experiments were carried out in 5 mmol L^{-1} $K_3[Fe(CN)_6]/K_4[Fe(CN)_6]$ + 0.1 mol L^{-1} KCl. Additionally, measurements for R_{ct}^{OH} were performed also in 0.1 mol L^{-1} KOH.

Electrochemical impedance spectroscopy (EIS) was used to determine charge transfer resistance for each catalyst in alkaline media as well as in redox system. In the Figure S5, the Nyquist plots of every catalyst registered in alkaline solution before chronoamperometric measurements as well as after linear sweep voltammetry stability studies were presented. Figure S6A and Figure S6B show impedance spectra utilized to determine charge transfer resistance using fitted equivalent circuit [3] and Randle's circuit, respectively [4].

Series of cyclic voltammograms with increasing scan rate from 5 to 500 mV s^{-1} were registered. Figure S7A shows a comparison of CV curves (ν = 100 mV s^{-1}) presenting well-shaped redox peaks of Fe(III)/Fe(II) ions registered on GCE sensor coated with appropriate catalysts with different bismuth contents. Based on iron oxidation and reduction peak potentials and the following formula: $(E_{ox}-E_{red})/0.059$ it is possible to calculate χ^0 factor. The closer this parameter comes to the theoretical value of $\chi^0=1$, the faster the electrode reaction occurs. There is also a visible dependence of the increase in the oxidation and reduction signals of iron ions with the increase of Bi concentration in the samples (Figure S7A) [5].

Figure S7B presents dependency between the anodic peak current (I_p) and the square root of the ν ($\nu^{1/2}$). Based on the Randles–Ševčík equation and the b coefficient obtained from the above-mentioned linear relationship, the value of the sensor's ECSA was calculated [5].

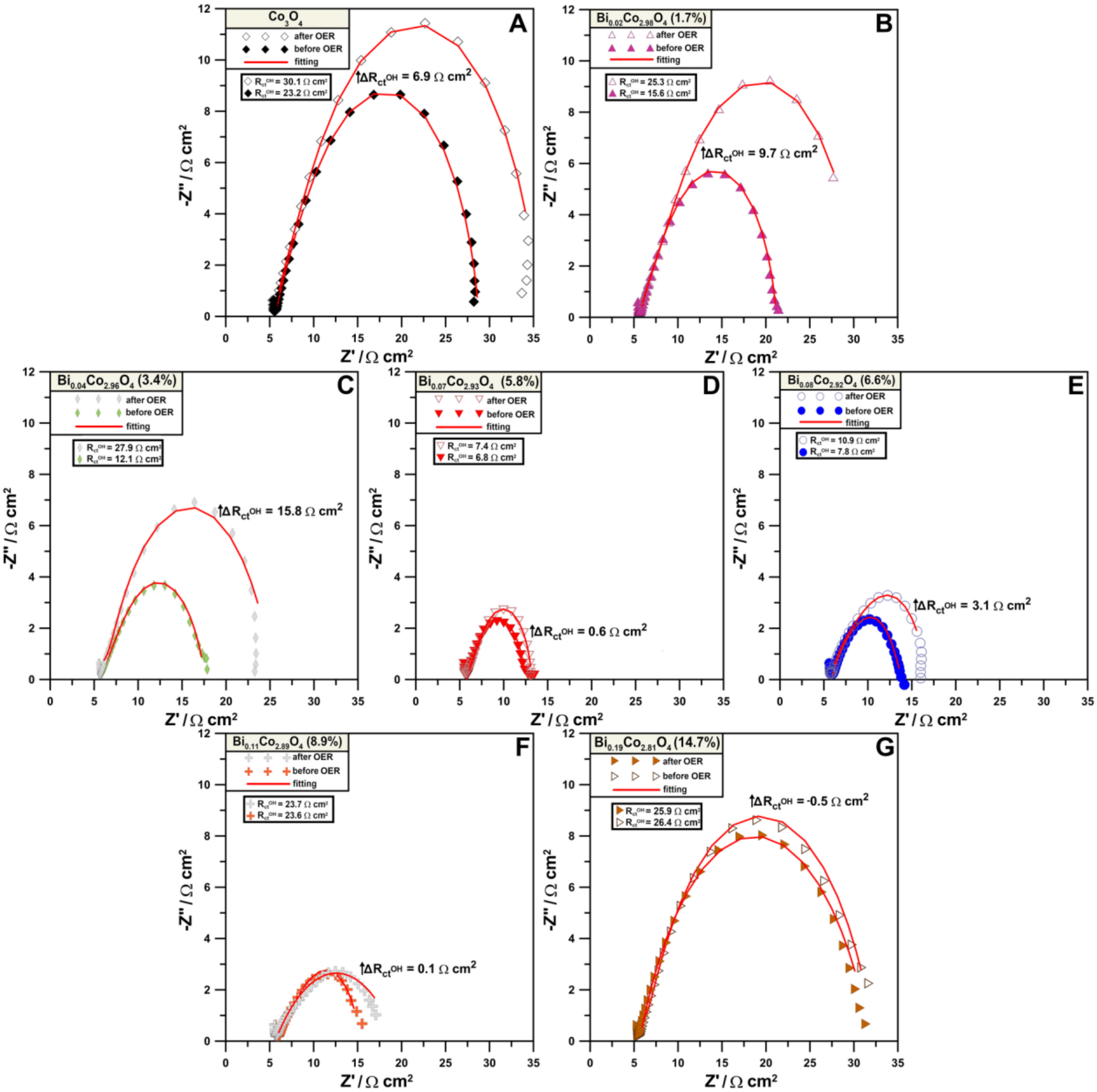

Figure S5. Nyquist plots obtained in 0.1 mol L^{-1} KOH solution (A-G). Cyclic voltammograms register for ν = 100 mV s^{-1} (C) and the dependency between the anodic peak current (I_p) and the square root of the ν ($\nu^{1/2}$) (D). Presented data were collected for all $Bi_xCo_{3-x}O_4$ samples.

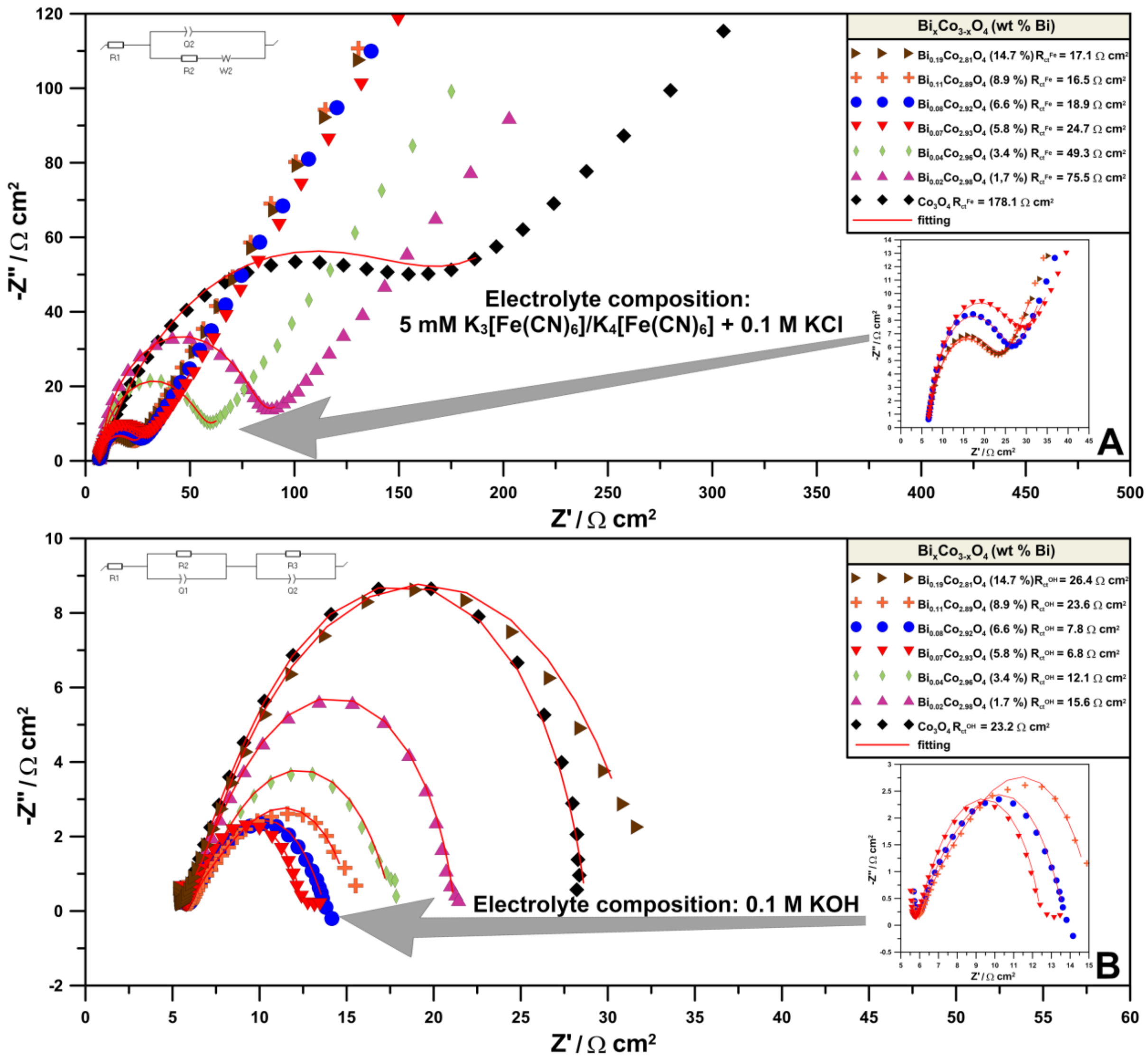

Figure S6. Nyquist plots obtained in alkaline (A) and redox system solution (B). Presented data were collected for all $Bi_xCo_{3-x}O_4$ samples.

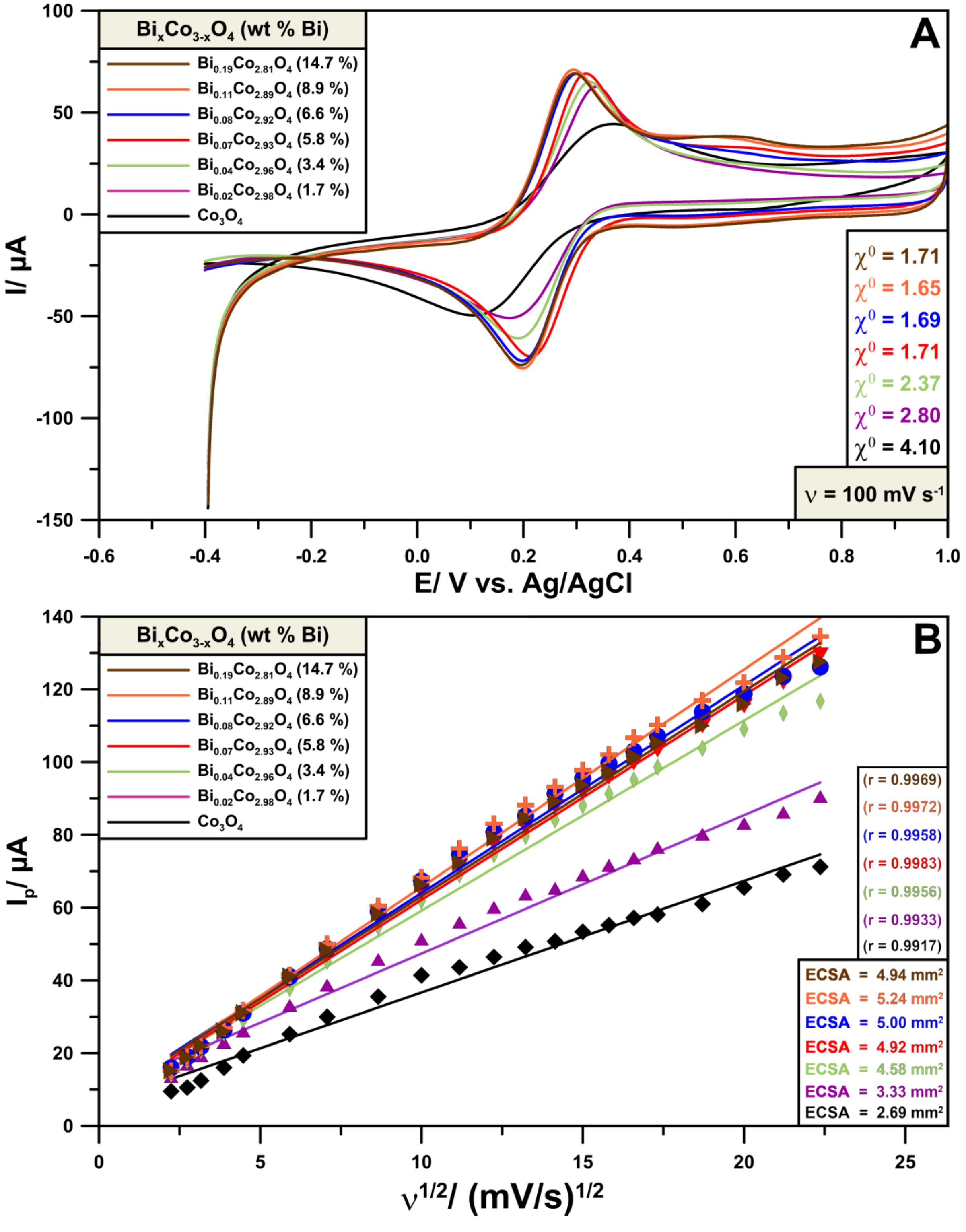

Figure S7. Cyclic voltammograms register in redox system solution for ν = 100 mV s^{-1} (A) and the dependency between the anodic peak current (I_p) and the square root of the ν ($\nu^{1/2}$) (B). Presented data were collected for all $Bi_xCo_{3-x}O_4$ samples.

Series of CV scans at a potential range of 1.2 − 1.3 V vs. RHE (0.35 – 0.45 V vs. Hg/HgO), with the ν = 2 - 12 mV s^{-1} (Figure S8A) was conducted to determine double-layer capacitance (C_{dl}). For each scan rate, the differential current (ΔI) measured at half of the potential window was calculated from following formula: $\Delta I = I_{forward} - I_{backward}$, where forward mean measurement conducted from 0.35 to 0.45 V and backward - in the opposite direction. Obtained values of ΔI for each scanning speed were used to prepare line charts for each catalyst (Figure S8B). Based on the value of the slope coefficient (b) from the following equation: ΔI [A] = bν [V s^{-1}] + a, which was divided by 2 (b/2), the C_{dl} [F] value was obtained [6].

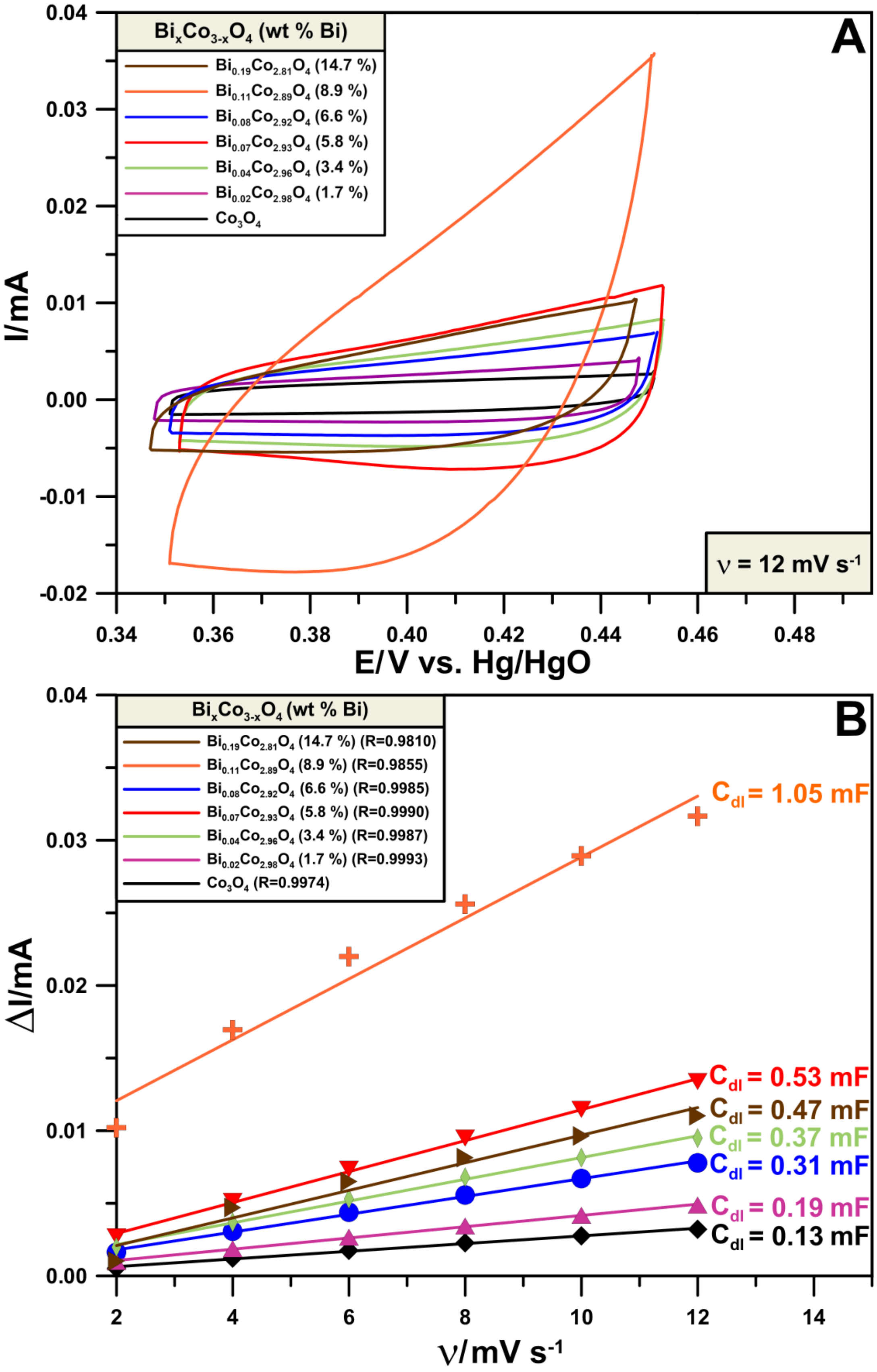

Figure S8. Comparison of GCE/$Bi_xCo_{3-x}O_4$ cyclic voltammograms (CV) registered for ν = 12 mV s^{-1} (A); double layer capacitance (C_{dl}) values calculated on the basis of relationship between differential current (ΔI) and scanning speed (ν = 2 - 12 mV s^{-1}) (B).

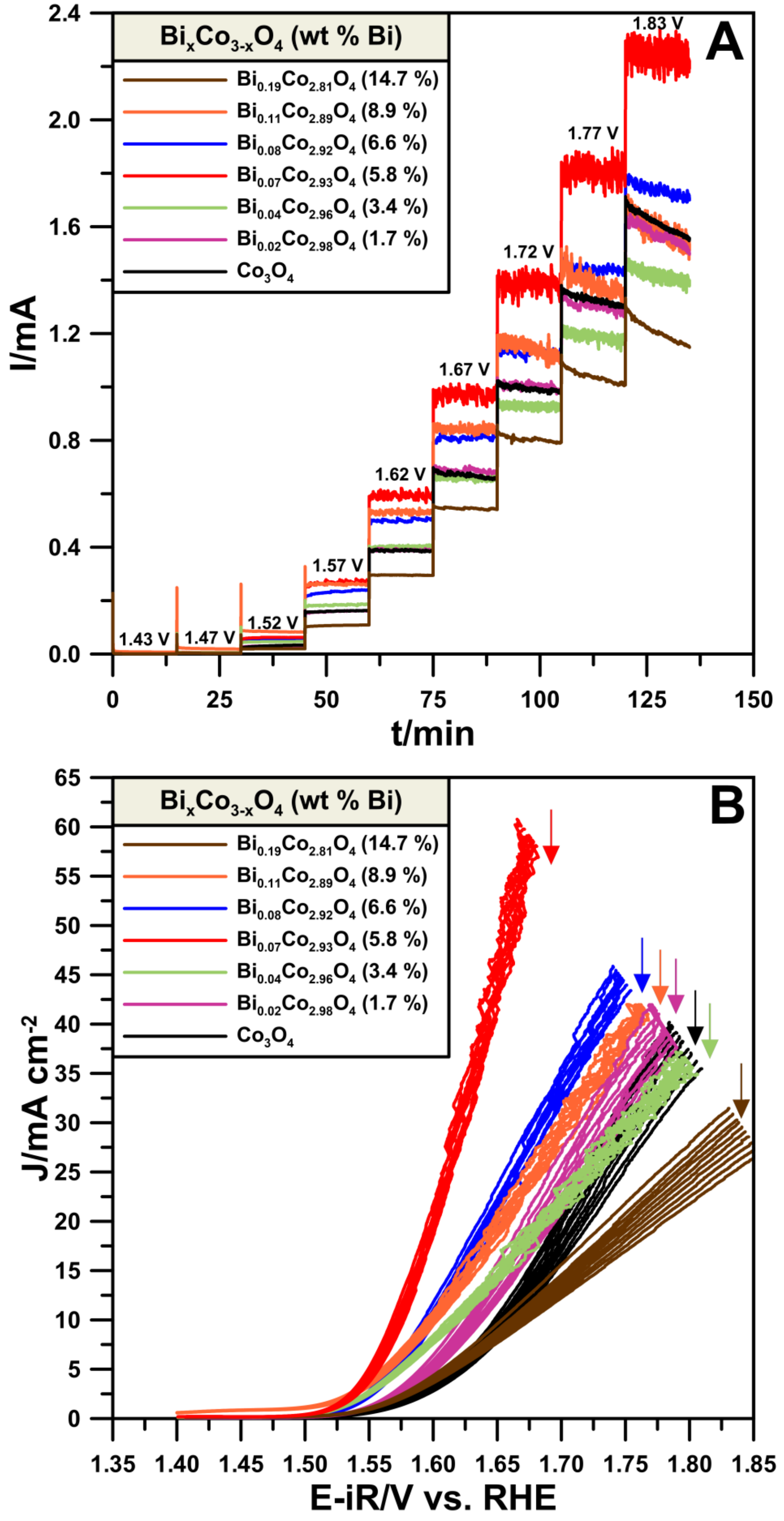

Figure S9. CA OER-activity (A) and LSV OER-stability (B) experiments.

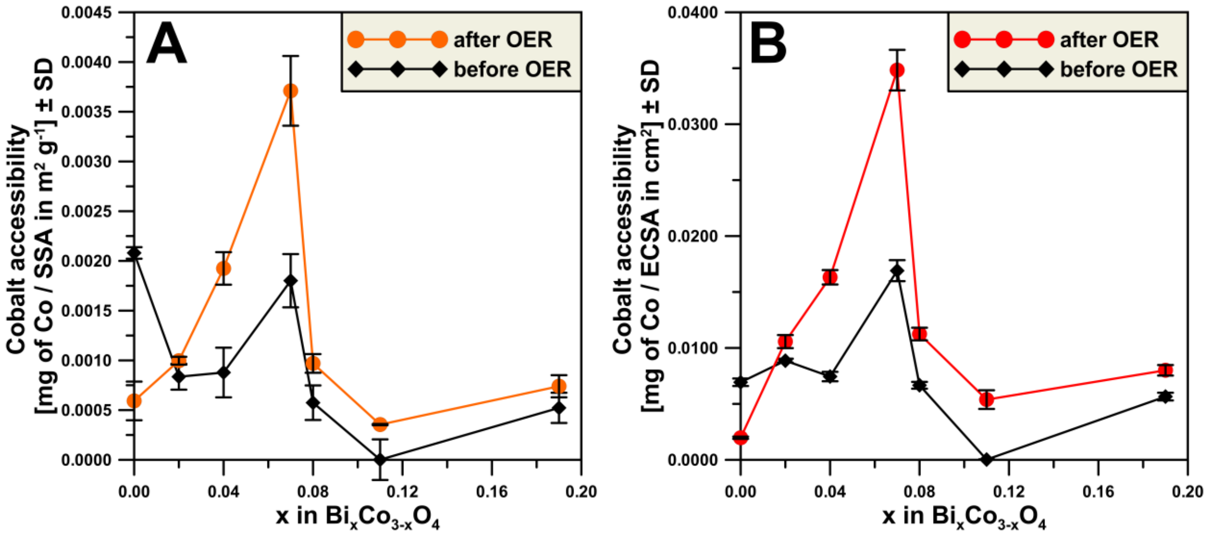

Figure S10. CV-based cobalt accessibility normalized using (A) specific surface area and (B) ECSA. SD were calculated for n = 3.

Section 5 – Density Functional Theory calculations

In this study, we used a Hubbard parameter value of U = 3.5 eV for Co cations, which has been shown to reproduce experimental results for Co_3O_4 accurately [7,8]. A thorough validation of this calculation scheme against experimental data was provided in our previous paper [9], where various bulk properties of cobalt oxide (including lattice parameters, band gap, and electronic and magnetic structures) were computed and analysed. The bulk unit cells of parent and Bi-doped cobalt spinel were derived by optimising the experimental cubic (1×1×1) unit cell (a = 8.08 Å) with $Co_{24}O_{32}$ stoichiometry (see Figure S11). The optimal cell volume was determined by fitting the energy-volume (E/V) data to the Birch-Murnaghan equation of state [10], with complete optimisation of all internal degrees of freedom to within an error margin of 10^{-4} eV Å^{-1}.

5.1 Bulk Co_3O_4 and $Bi_xCo_{3-x}O_4$ structure

The cobalt spinel oxide (Co_3O_4) structure ($Fd3m$ symmetry space group) with the unit-cell containing 8 formula units ($Co_{24}O_{36}$ stoichiometry) is shown in Figure S11a. The oxygen anions (indicated red) occupy 32e Wycoff positions, thus forming an FCC lattice in which one-eighth of the available tetrahedral sites (8a positions) are occupied by divalent cations (purple), whereas half of the octahedral sites (16d) host trivalent cations (blue), in line with normal-spinel structure. The optimized lattice constant, determined using the Birch-Murnaghan equation of state, equals 8.149 Å, and the interionic distances within tetrahedral and octahedral sites (Co^{8a}-O and Co^{16d}-O bonds) are 1.974 Å and 1.926 Å, respectively. Both cationic positions are possible loci for bismuth substitution thus we compared two models (see Figure S11b and S11c) corresponding to tetrahedral and octahedral Bi substitution. The GGA+U modeling confirmed that in both cases bismuth adopts its optimal oxidation state (Bi^{3+}) and due to its larger ionic radius compared to cobalt ions, it slightly increases the Co_3O_4 optimal lattice constant (Figure S11b and S11c). Energy analysis indicates that bismuth preferentially occupies octahedral sites, replacing an isovalent cobalt ion without affecting the redox state of the spinel matrix. In the less energetically favorable (ΔE = 0.45 eV) tetrahedral (8a) substitution, replacing Co^{2+} with hypervalent Bi^{3+} generates an electron hole which is localized on the adjacent octahedral cobalt.

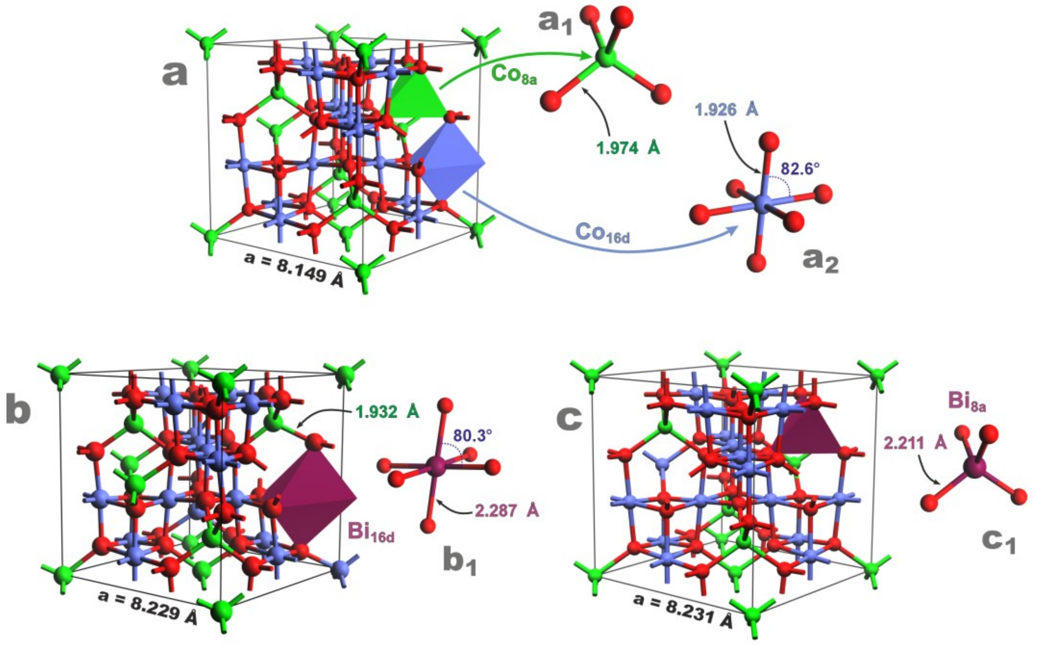

Figure S11. Bulk structure of cobalt spinel oxide (a); bismuth-doped derivatives hosting Bi in the octahedral (16d) (b) and tetrahedral (8a) (c) cationic positions.

5.2 Slab models of Co_3O_4 and $Bi_xCo_{3-x}O_4$ (100) surfaces

Surface slabs representing the most stable (100) surface of cobalt spinel were created by cleaving the optimised bulk structures along the normal [100] direction to form large supercells containing approximately 10 atomic layers with a 25 Å vacuum space. The slab models of pure and bismuth-doped spinel are shown in Figure S12a and S12b, respectively. The top and bottom slab terminations were constructed identically to avoid developing unrealistic dipole moments within the cell. Relaxation of atomic positions in the top four layers was conducted to ensure forces acting upon the ions were below 1×10^{-2} eV Å^{-1}.

The geometry optimization was applied to the upper and lower layers of the slab model, while the positions of the central atoms were fixed (indicated by the appropriate background color). Perspective views of the corresponding (1×1) surface elements after geometric relaxation are presented in Figures S12a_1 and S12b_1.

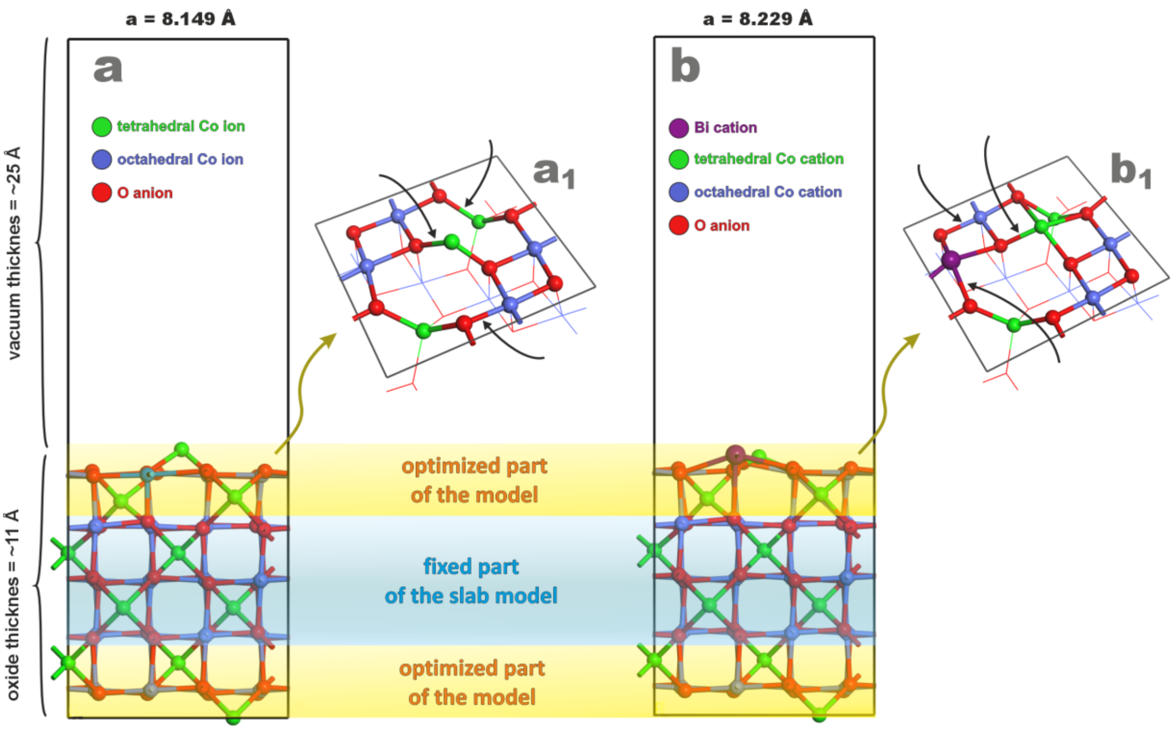

Figure S12. Slab models representing (100) surface of parent cobalt spinel oxide (a) and bismuth-doped Co_3O_4 (b).

In the case of parent cobalt spinel, the cationic composition of the (1 × 1) surface element consists of pentacoordinated Co_{16d} ions (colored blue), one protruding tetrahedral Co_{8d} of lowered coordination (2-fold), and two concealed tetrahedral (colored green) cobalt ions with full coordination situated in the first sublayer. The surface anionic composition includes 8 oxide ions, among which 6 exhibit reduced 3-fold coordination, while the remaining two maintain their bulk 4-fold coordination. As indicated by Figure S12a, the surface reconstruction is minimal. Both Co_{16d}–O and Co_{8a}–O bonds are slightly shorter than in the bulk, however, the tetrahedral angle of the recessed Co_{8a} is significantly larger than in the bulk (117° vs. 109°). In the case of the bismuth-doped surface (Figure S12b), two dominant optimization effects are observed. The M-O bonds change their length significantly, and the protruding cobalt atom migrates to the empty 16c site. This effect, known as off-stoichiometry, has been previously described in detail [9].

5.3 OER free energy diagrams

For the construction of OER free energy diagrams, the methodology developed by Norskøv *et al*. was employed [11–13] with the assumption that the overall process occurs in four single-electron steps (Equations s1-s4), characterised by corresponding free enthalpies ($\Delta G_{1\text{-}4}$):

(Equation s1) $H_2O_{(l)} + * \leftrightarrow HO* + H^+ + e^-$ $\Delta G_1 = \Delta G_{HO*} - \Delta G_{H2O(l)} - eU + k_bT\ln(a_{H+})$

(Equation s2) $HO* \leftrightarrow O* + H^+ + e^-$ $\Delta G_2 = \Delta G_{O*} - \Delta G_{HO*} - eU + k_bT\ln(a_{H+})$

(Equation s3) $O* + H_2O_{(l)} \leftrightarrow HOO* + H^+ + e^-$ $\Delta G_3 = \Delta G_{HOO*} - \Delta G_{O*} - eU + k_bT\ln(a_{H+})$

(Equation s4) $HOO* \leftrightarrow O_{2(g)} + H^+ + e^-$ $\Delta G_4 = \Delta G_{O2} - \Delta G_{HOO*} - eU + k_bT\ln(a_{H+})$

The initial step involves water splitting at the electrocatalyst active site, forming an HO* intermediate (Equation s1). The subsequent step entails oxidising the HO* species to O* (Equation s2). The third step involves second water dissociation atop oxygen species, forming OOH* (Equation s3). The process concludes with the evolution of oxygen (Equation s4).

The derivation of the $\Delta G_{1\text{-}4}$ values was based on the following assumptions: (1) the influence of liquid water was implicitly considered by using it as the reference state; (2) the use of computational standard hydrogen electrode (SHE) allowed to substitute the proton and the electron with half a hydrogen molecule [14]; (3) the theoretical overpotential remains independent of pH or specific potential values, as the free energies vary similarly with pH and U; (4) the free energy analysis was conducted under standard conditions (pH = 0, T = 298.15 K) and U = 0 eV. The calculation of the free energies ($\Delta G_{1\text{-}4}$) requires the calculation of the electronic (DFT) energies of ORR intermediates stabilization (see below, Figure S13) and appropriate changes related to the entropic term of the system associated with the adsorption of the involved molecules. Assuming that the free enthalpy of the support does not change significantly upon interaction with ORR reactants and intermediates (H_2O, O_2, H_2*O, *OH, H^+), only the changes in the chemical potentials of the involved molecular/atomic species are significant. They were calculated using corresponding entropic corrections and Zero Point Energies obtained thanks to statistical thermodynamics, following the procedure defined in detail by Man et al. [11].

An essential parameter that can be deduced from the free energy diagram is the magnitude of the potential-determining step, which is closely related to the activity of the electrocatalyst (Figure S13 and associated text). Figure S13 illustrates the energy diagram for an ideal oxygen evolution catalyst to facilitate water oxidation just above the equilibrium potential. This necessitates that all four charge transfer steps have free energies of reaction of equal magnitude at zero potential (4.92 eV/4 = 1.23 eV) and is equivalent to all free energies of consecutive steps being zero at the equilibrium potential of 1.23 V (see orange line). A catalyst meeting this criterion is thermochemically ideal and real catalysts should be as close to this characteristic as possible.

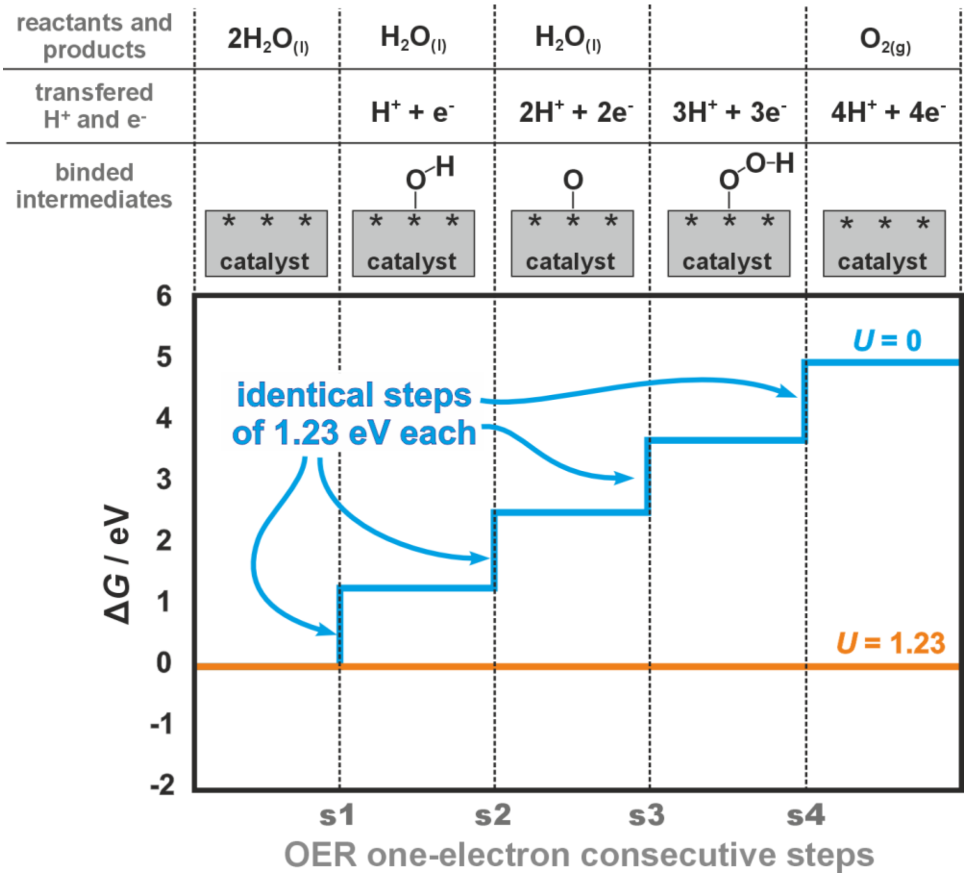

Figure S13. Standard free energy diagram for the OER process at zero potential ($U = 0$, blue line), and equilibrium potential for oxygen evolution ($U = 1.23$ eV, orange line), over the ideal catalyst (at pH = 0 and $T = 298$ K).

5.4 The potential determining step of OER/ORR processes

The potential-determining step of electrochemical OER and ORR processes (explored in works of the Norskov group [13,15]) is the last step on the free energy diagram to become downhill as the potential increases, corresponding to the specific reaction step in the four-step mechanism with the largest ΔG. Free energy changes along the reaction pathway are directly correlated with the binding energies of intermediates $\Delta E_{(X)}^{DFT}$ (where X stands for OH, O, and OOH), which have been calculated according to the following expressions (Equations s5-s7):

(Equation S5) $$\Delta E_{(OH)}^{DFT} = \left(E_{*OH}^{DFT} + \frac{1}{2}E_{H2(g)}^{DFT}\right) - \left(E_{*}^{DFT} + E_{H2O(l)}^{DFT}\right)$$

(Equation S6) $$\Delta E_{(O)}^{DFT} = \left(E_{*O}^{DFT} + 2E_{H2(g)}^{DFT}\right) - \left(E_{*}^{DFT} + E_{H2O(l)}^{DFT}\right)$$

(Equation S7) $$\Delta E_{(O)}^{DFT} = \left(E_{*OOH}^{DFT} + 3/2E_{H2(g)}^{DFT}\right) - \left(E_{*}^{DFT} + 2E_{H2O(l)}^{DFT}\right)$$

where: E_{*}^{DFT}, E_{*OH}^{DFT}, E_{*O}^{DFT}, E_{*OOH}^{DFT} stand for DFT energy of bare slab model, and slab models with OH, O, and OOH intermediates adsorbed, respectively, whereas $E_{H2O(l)}^{DFT}$ and $E_{H2(g)}^{DFT}$ denotes DFT energies of liquid water and gaseous hydrogen.

5.5 OER intermediates adsorption DFT studies

The DFT-optimized geometries of OER intermediates bound to different active sites of Co_3O_4 and $Bi_xCo_{3-x}O_4$ catalysts are presented in Figure S14. In the first case, these were the tetrahedral (Figure 14a) and octahedral (Figure 14b$_1$-b$_3$) cobalt centers (see Figures 14a$_1$-a$_3$ and 14b$_1$-b$_3$, respectively), whereas in the second case (according to the established energetics of cobalt substitution, see Figure S11 and S12), the bismuth center in the 16d position (Figure S14c$_1$-c$_3$) and the neighboring Co_{16d} center (Figure S14d$_1$-S14d$_3$).

An inspection of the presented structures indicates that adsorption occurs in a vertical η^1 configuration through the oxygen atom in each case, and the only significant differences concern the Co–O bond lengths. The shortest bonds characterize the Co_{8a} centers on the pure spinel (which can be attributed to their strong coordinative unsaturation; see Figure S14a$_1$-S14a$_3$), while the longest bonds are found in the Bi_{16d} centers (here, the decisive factor is the large ionic radius of Bi, see Figure S14c$_1$-S14c$_3$). Population analysis indicates that in each case, the ad-species accumulate about 1 electron, originating from the surface octahedral Co_{16d} cations, which are oxidized to Co^{4+} (such redox behavior of cobalt spinel has been previously described in detail [16]). As a result of this charge transfer, hydroxyl groups (OH^-, Figure 14a$_1$-14d$_1$), metal-oxo type adducts (M-O^-, Figure 14a$_2$-14d$_2$), and hydroperoxyl anions (OOH^-, Figure 14a$_3$-14d$_3$) are formed on the catalysts surface.

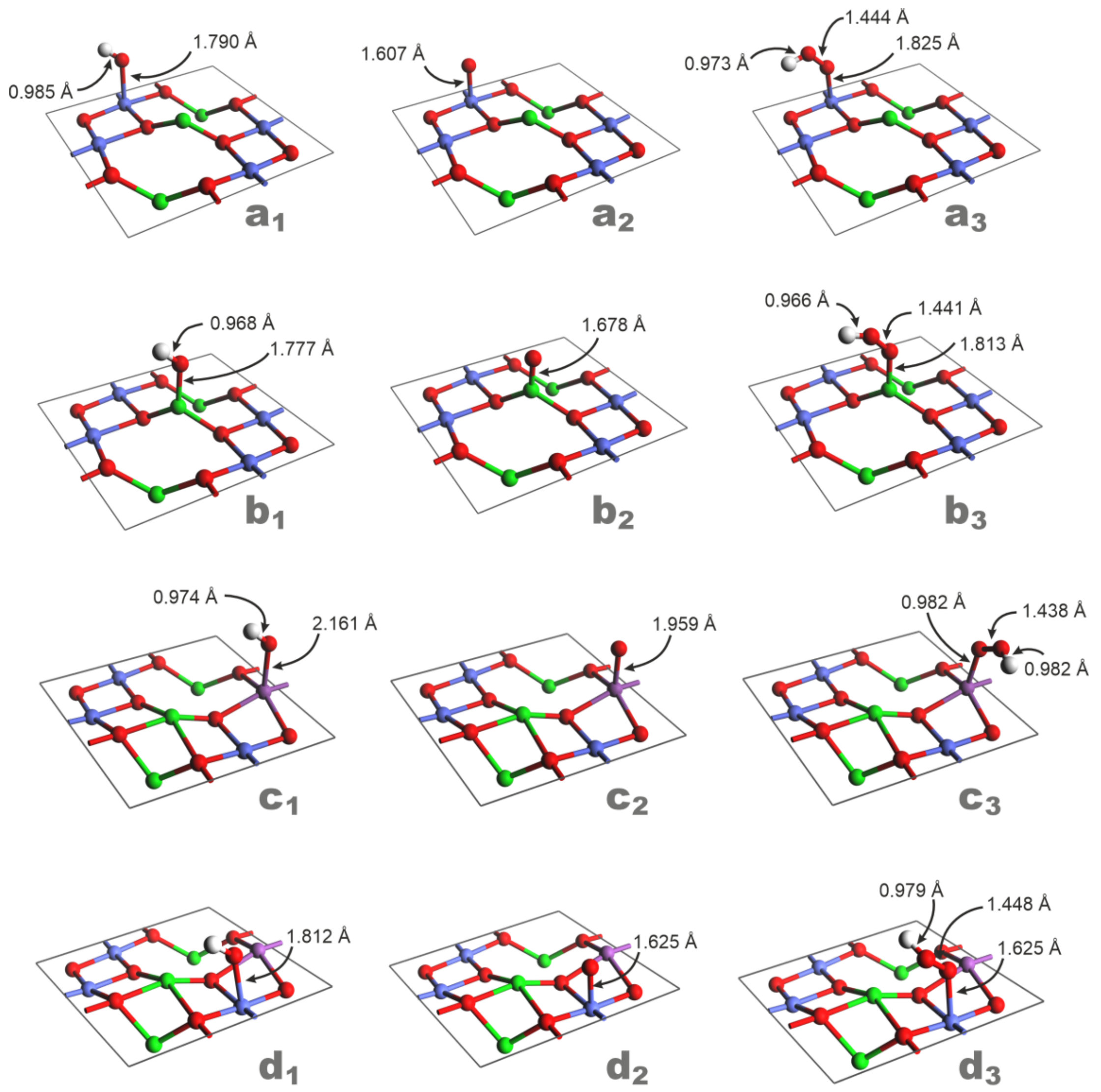

Figure S14. The DFT-optimized geometries of OER intermediates bound to Co_{8a} (a_1-a_3) and Co_{16d} (b_1-b_3) surface sites of bare Co_3O_4 (100) surface, and to Bi_{16d} (c_1-c_3) and Co_{16d} (d_1-d_3) surface sites of $Bi_xCo_{3-x}O_4$.

5.6 Projected electronic density of states

The total projected electronic density of states (DOS) for bulk Co_3O_4 and partial DOS for Co^{oct} and Co^{tet} cations are illustrated in Figures S15a_1, a_2, and a_3 respectively. For our modelling we choose slightly more stable antiferromagnetic configuration of Co^{tet} sublattice, thus total DOS is highly symmetric with virtually no spin polarization visible. The pDOS for Co^{oct} ions is nearly symmetric (Figure S15a_2), aligning well with the zero magnetic moment characteristic of spin-paired Co^{3+} ions ($t_{2g}^6e_g^0$ configuration, O_h symmetry). The t_{2g} states in Co^{oct} cluster near the upper portion of the valence band (–1.5 eV), while the e_g states are centered around 2.5 eV, demonstrating a clear crystal field splitting. The magnetic nature of Co^{tet} cations which T_h environment which results in $e^4t_2^3$ configuration is clearly visible in both

plots presented in Figure S15a3. The graphs illustrate two subsets belonging to the tetrahedral cationic sublattice of cobalt, differing in the direction of the net spin on divalent cations (arranged antiferromagnetically).

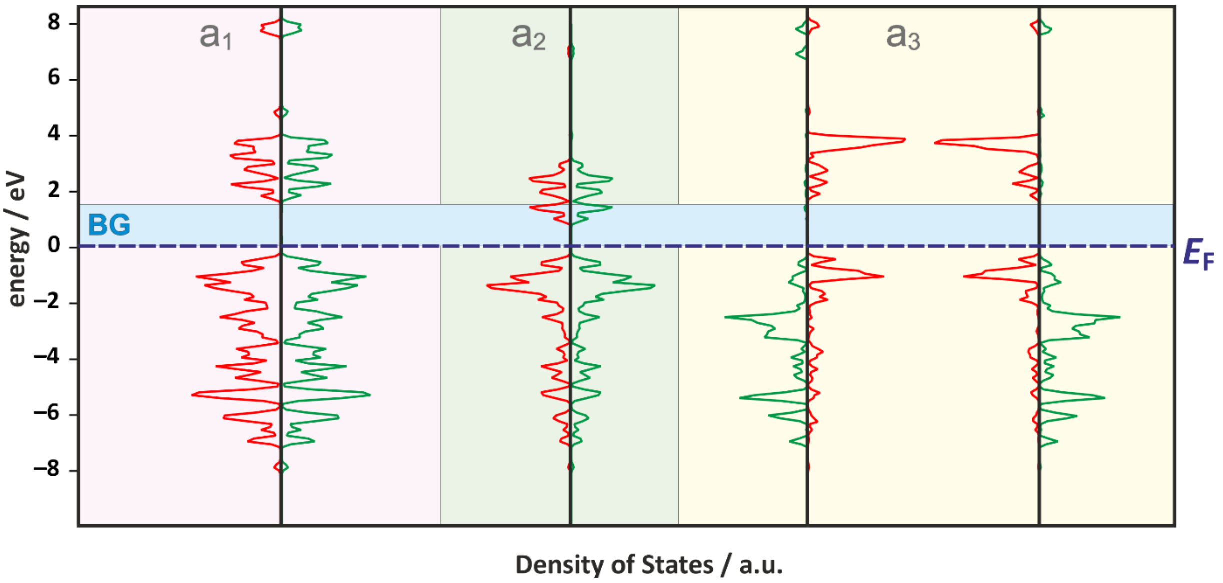

Figure S15. The total projected electronic density of states (DOS) for bulk Co_3O_4 (a_1) and partial DOS for Co^{oct} (a_2) and Co^{tet} (a_3) cations (with opposite directions of magnetic moments). The majority(α) and minority(β)-spin states are marked red and green, respectively.

In the first case, the majority-spin 3*d* states (green line) are split into the *e* states located at about –2 eV, whereas the t_2 states form a triplet peak at about –6 eV (relative to the Fermi level). The occupied minority-spin *e* states are positioned near the top of the valence band (–0.5 eV), while the unoccupied t_2 states dominate the lower conduction band at 3.2 eV. In the second case, the spin arrangement is analogous, but the α and β spins switch roles. The valence band maximum comprises contributions from both tetrahedral Co-3d and O-2p states, while the conduction band minimum is predominantly derived from octahedral Co-3d states. The direct Γ–Γ band gap calculated using the DFT+U approach is 1.73 eV, closely matching the experimental value of 1.6 eV and aligning with previous theoretical results [17]. The calculated X-X transition energy was equal to 1.23 eV, and an indirect Γ-X transition to 1.51 eV, in line with optical measurements, where a range of allowed and forbidden transitions in the region from 1.1 to 2.06 eV have been observed [18].

The experimentally reproduced electron-spin structure of cobalt spinel, calculated with high accuracy, unequivocally indicates a well-chosen parameterization of the DFT method.

References

[1] M. Lofek, A. Ryłko, G. Grzybek, A. Ejsmont, T. Darvishzad, J. Goscianska, A. Kotarba, P. Stelmachowski, Electrocatalytic activity in the oxygen evolution reaction of nitrogen-doped mesoporous carbon-supported cobalt oxide nanoparticles, Catal. Today 441 (2024) 114878. https://doi.org/10.1016/j.cattod.2024.114878.

[2] G. Spinolo, S. Ardizzone, S. Trasatti, Surface characterization of Co3O4 electrodes prepared by the sol-gel method, J. Electroanal. Chem. 423 (1997) 49–57. https://doi.org/10.1016/S0022-0728(96)04841-3.

[3] G.A. Gebreslase, D. Sebastián, M.V. Martínez-Huerta, M.J. Lázaro, Nitrogen-doped carbon decorated-Ni3Fe@Fe3O4 electrocatalyst with enhanced oxygen evolution reaction performance, J. Electroanal. Chem. 925 (2022) 116887. https://doi.org/10.1016/j.jelechem.2022.116887.

[4] A.Ch. Lazanas, M.I. Prodromidis, Electrochemical Impedance Spectroscopy─A Tutorial, ACS Meas. Sci. Au 3 (2023) 162–193. https://doi.org/10.1021/acsmeasuresciau.2c00070.

[5] F. Beck, Cyclic voltammetry—simulation and analysis of reaction mechanisms. By David K. Gosser, Jr., VCH, New York 1993, xi, 154 pp., hardcover, DM 124.00, ISBN 3-527-28226-2, disks included (5 1/4″ and 3 1/2″), Electroanalysis 7 (1995) 298–298. https://doi.org/10.1002/elan.1140070324.

[6] S. Anantharaj, S.R. Ede, K. Karthick, S. Sam Sankar, K. Sangeetha, P.E. Karthik, S. Kundu, Precision and correctness in the evaluation of electrocatalytic water splitting: revisiting activity parameters with a critical assessment, Energy Environ. Sci. 11 (2018) 744–771. https://doi.org/10.1039/C7EE03457A.

[7] L. Wang, T. Maxisch, G. Ceder, Oxidation energies of transition metal oxides within the GGA + U framework, Phys. Rev. B 73 (2006) 195107. https://doi.org/10.1103/PhysRevB.73.195107.

[8] M. García-Mota, M. Bajdich, V. Viswanathan, A. Vojvodic, A.T. Bell, J.K. Nørskov, Importance of Correlation in Determining Electrocatalytic Oxygen Evolution Activity on Cobalt Oxides, J. Phys. Chem. C 116 (2012) 21077–21082. https://doi.org/10.1021/jp306303y.

[9] F. Zasada, W. Piskorz, Z. Sojka, Cobalt Spinel at Various Redox Conditions: DFT+U Investigations into the Structure and Surface Thermodynamics of the (100) Facet, J. Phys. Chem. C 119 (2015) 19180–19191. https://doi.org/10.1021/acs.jpcc.5b05136.

[10] F.D. Murnaghan, The Compressibility of Media under Extreme Pressures, Proc. Natl. Acad. Sci. 30 (1944) 244–247. https://doi.org/10.1073/pnas.30.9.244.

[11] I.C. Man, H. Su, F. Calle-Vallejo, H.A. Hansen, J.I. Martínez, N.G. Inoglu, J. Kitchin, T.F. Jaramillo, J.K. Nørskov, J. Rossmeisl, Universality in Oxygen Evolution Electrocatalysis on Oxide Surfaces, ChemCatChem 3 (2011) 1159–1165. https://doi.org/10.1002/cctc.201000397.

[12] J. Rossmeisl, Z.-W. Qu, H. Zhu, G.-J. Kroes, J.K. Nørskov, Electrolysis of water on oxide surfaces, J. Electroanal. Chem. 607 (2007) 83–89. https://doi.org/10.1016/j.jelechem.2006.11.008.

[13] J.K. Nørskov, J. Rossmeisl, A. Logadottir, L. Lindqvist, J.R. Kitchin, T. Bligaard, H. Jónsson, Origin of the Overpotential for Oxygen Reduction at a Fuel-Cell Cathode, J. Phys. Chem. B 108 (2004) 17886–17892. https://doi.org/10.1021/jp047349j.

[14] M. Methfessel, A.T. Paxton, High-precision sampling for Brillouin-zone integration in metals, Phys. Rev. B 40 (1989) 3616–3621. https://doi.org/10.1103/PhysRevB.40.3616.

[15] J. Rossmeisl, A. Logadottir, J.K. Nørskov, Electrolysis of water on (oxidized) metal surfaces, Chem. Phys. 319 (2005) 178–184. https://doi.org/10.1016/j.chemphys.2005.05.038.

[16] F. Zasada, W. Piskorz, J. Janas, J. Gryboś, P. Indyka, Z. Sojka, Reactive Oxygen Species on the (100) Facet of Cobalt Spinel Nanocatalyst and their Relevance in $^{16}O_2$ / $^{18}O_2$ Isotopic Exchange, *de* N_2O, and *de* CH_4 Processes—A Theoretical and Experimental Account, ACS Catal. 5 (2015) 6879–6892. https://doi.org/10.1021/acscatal.5b01900.
[17] P.S. Patil, L.D. Kadam, C.D. Lokhande, Preparation and characterization of spray pyrolysed cobalt oxide thin films, Thin Solid Films 272 (1996) 29–32. https://doi.org/10.1016/0040-6090(95)06907-0.
[18] S. Thota, A. Kumar, J. Kumar, Optical, electrical and magnetic properties of Co3O4 nanocrystallites obtained by thermal decomposition of sol–gel derived oxalates, Mater. Sci. Eng. B 164 (2009) 30–37. https://doi.org/10.1016/j.mseb.2009.06.002.
